# The development of a direct co-culture-based model for diabetic foot ulcer mimicking inflammation and impaired phagocytosis

**DOI:** 10.1007/s44164-024-00080-5

**Published:** 2025-04-14

**Authors:** Mirella Ejiugwo, Yury Rochev, Georgina Gethin, Gerard O’Connor

**Affiliations:** 1 School of Natural Sciences, University of Galway, University Road, Galway, H91 TK33 Ireland; 2 School of Nursing and Midwifery, Aras Moyola, University of Galway, University Road, Galway, H91 TK33 Ireland

**Keywords:** Diabetic foot ulcer, Chronic wounds, Inflammation, Impaired phagocytosis, In vitro wound model

## Abstract

**Purpose:**

Diabetic foot ulcers (DFU) are characterized by delayed healing and high infection rates. DFU affect approximately 25% of individuals with diabetes. Secondary to hyperglycaemia, both chronic inflammation and defective phagocytosis have been identified as contributing factors to the non-healing status of DFU. Both inflammation and defective phagocytosis in DFU were sought to be modelled in vitro using pHRODO bioparticles for the first time. The pHRODO bioparticles, popularly used as phagocytic cargos, are chemically killed microorganisms conjugated to the pH-sensitive pHRODO dye that solely fluoresces within the acidic lysosomes where phagocytosis occurs.

**Methods:**

The in vitro DFU model was developed by identifying which ratio of diabetic fibroblasts to THP-1-derived Mɸ, choice of pHRODO bioparticles, FBS concentration, and oxygen level exhibited both significant inflammation and reduced phagocytic ability. Inflammation was confirmed via simultaneous TNF-α and MCP-1 release by direct co-cultures of diabetic fibroblasts and THP-1-derived macrophages (Mɸ) following pHRODO bioparticle exposure using ELISA. Phagocytic activity, derived from the emitted fluorescence of ingested pHRODO bioparticles within acidic lysosomes, was quantified using an automated, whole-well, fluorescent imaging system. The kinase Bay 11–7085, shown to stimulate phagocytosis previously, was used to verify the usefulness of the developed in vitro DFU model.

**Results:**

Inflammation and reduced phagocytic activity were observed maximally for a 1:4 ratio of diabetic dermal fibroblasts to THP-1-derived Mɸ upon 4-h incubation with 200 µg/ml pHRODO green *Staphylococcus aureus* bioparticles under hypoxia (2% oxygen) and low nutrient level (2% fetal bovine serum)—compared with the in vitro healthy wound model. When co-delivered with Bay 11–7085, significant increased uptake of pHRODO green *S. aureus* bioparticles was observed in the in vitro DFU model.

**Conclusion:**

Optimized parameters for modeling inflammation and reduced phagocytic activity in DFU in vitro were identified. Modulating inflammation could be useful in stimulating phagocytosis in DFU based on the positive effect of Bay 11–7085 on the in vitro DFU model. This finding paves the way for screening and re-purposing immunomodulatory drugs to stimulate phagocytosis in DFU.

**Graphical abstract:**

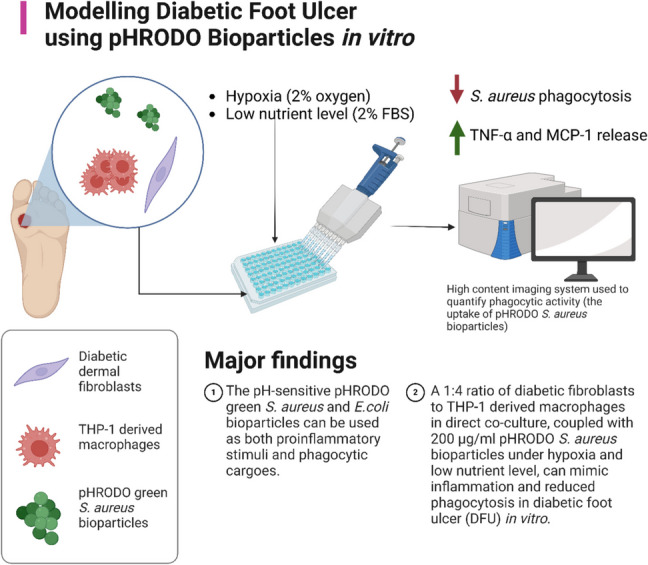

**Supplementary Information:**

The online version contains supplementary material available at 10.1007/s44164-024-00080-5.

## Introduction

Worldwide, 1 in 11 adults between 20 and 79 years of age has diabetes mellitus (DM) [1–3]. It has been estimated that 578 million individuals will have diabetes by 2030 and 700 million by 2045 [1].

DM is a complex, non-curable condition that is associated with multiple complications including retinopathy, renal disease, peripheral vascular disease and foot disease [4, 5]. One out of four persons with DM has a lifetime risk of developing a foot ulcer. DFU can result from the contributing factors of neuropathy, ischaemia, foot deformities, trauma and peripheral arterial occlusive diseases [6, 7]. Multiple factors contribute to delayed healing of DFU, including the poor transition of macrophages (Mɸ) from a pro-inflammatory (non-healing) to an anti-inflammatory (pro-healing) phenotype [8]. The increased ratio of pro-inflammatory to anti-inflammatory Mɸ within the diabetic wound bed is marked by high levels of pro-inflammatory mediators like tumor necrosis factor-alpha (TNF-α), monocyte chemoattractant protein-1 (MCP-1) and interleukin-6 (IL-6) [9, 10]. These proinflammatory factors, coupled with reduced bactericidal activity of phagocytes, contribute to poor phagocytic and wound healing abilities in DFU [9].

Phagocytosis can be described as the sensing and subsequent ingestion of foreign particles larger than 0.5 μm, found in the extracellular space, by phagocytes [11]. Examples of foreign particles include microbes, apoptotic and necrotic cells. Upon particle ingestion, intracellular phagosomes, which are specialized vacuoles, are formed. Eventually, the phagosomes fuse with lysosomes (pH 4.5) to become phagolysosomes (phagosome maturation) [12]. Phagolysosomes contain anti-microbial components including hydrochloric acid and hydrolytic enzymes, which are responsible for the breakdown of the ingested foreign particles [11].

Phagocytosis has been reported to be defective in individuals with diabetes coupled with increased risk of infections [10, 13, 14]. Impaired phagocytosis results in infection, biofilm formation, and accumulation of dead cells and thriving bacteria within the diabetic wound bed [14]. These events result in significant inflammation (increased pro-inflammatory cytokine levels), decreased collagen deposition, increased extracellular matrix (ECM) breakdown, reduced growth factor production, impaired cell proliferation and migration, poor angiogenesis and reduced wound closure [13, 15, 16]. A positive feedback pro-inflammatory cycle ensues and, consequently, the diabetic wound remains “open,” stuck in inflammation [17, 18]. In fact, between 15 and 50% of DFU cases get infected and, hence, require either partial or complete amputation of the affected lower limb or region, if not treated timely [19]. Following amputation, there is 50% probability of death occurring within 5 years [20].

In complying with the 3R’s (i.e., reduction, refinement, and replacement) associated with the use of animals for research, developing alternative in vitro, yet robust, systems for drug screening to identify effective hits for DFU treatment are desirable [21]. Till date, 3D in vitro models have been employed for studying DFU successfully, especially re-epithelialization [22–24] and angiogenesis [22, 25]. However, despite the crucial role that reduced phagocytosis plays in preventing tissue repair in DFU, it is yet to be considered within in vitro DFU models, especially in those incorporating either Mɸ or monocytes [24, 26].

Phagocytosis assays have been popularly used to evaluate the clearance ability of immune cells, especially Mɸ and neutrophils. The major difference amongst in vitro phagocytosis assays lies in the nature of the used phagocytic cargo. This latter could fall in either of two categories: (1) model cells (e.g., bacteria, fungi) or (2) protein-coated beads (e.g., phosphatidylserine, antibodies). Using model cells provides the physiological complexity of phagocytic targets in studying phagocytosis. On the other hand, protein-coated beads, although they lack the ability to deform, are useful for studying the involvement of a particular ligand or receptor in phagocytosis [27].

Most commonly for in vitro phagocytosis assays, latex beads are labelled with fluorescent dyes (e.g., Alexa Fluor 488 NHS ester) and used to challenge cells of interest. Fluorescence derived from membrane-bound beads is quenched extracellularly using trypan blue prior to detecting ingested beads using either fluorescence microscopy or flow cytometry [28, 29].

One of the most advanced means for quantifying phagocytic activity in vitro is the exposure of phagocytes to pHRODO™ bioparticles [30, 31]. The pHRODO™ bioparticles consist of chemically killed whole microorganisms (e.g., *Escherichia coli* and *Staphylococcus aureus*) conjugated to the pH-sensitive and fluorogenic pHRODO dye. The pHRODO bioparticles do not fluoresce at neutral pH. Following successful uptake of the pHRODO particles into the acidic phagolysosomal compartments of phagocytes, the emitted fluorescence intensity increases (Supplementary Fig. 1). Hence, upon addition to cells of interest, the pHRODO bioparticles require no washing and quenching steps prior to imaging, compared with other types of phagocytic cargos.

The pHRODO bioparticles have been used in several published works till date and the arising fluorescence (indicative of successful bioparticle uptake) can be measured using different approaches, ranging from conventional fluorescence microscopy, spectrofluorimetry, flow cytometry to high-throughput screening [32–35].

While pHRODO bioparticles have been adopted for both in in vivo and in vitro phagocytosis studies, and reported accordingly [30, 33–40], the literature has yet to describe the *proinflammatory use* of these bioconjugates (given their bacterial nature) for disease modelling. Current commercially available pHRODO products are conjugated to either *E. coli*, *S. aureus* or zymosan. Bacterial and fungal microorganisms are known to elicit an immune response, resulting in significant proinflammatory cytokine release [41]. Hence, the pHRODO bioparticles could function as alternatives to conventional proinflammatory stimuli such as lipopolysaccharide (LPS) from Gram-negative bacteria, and lipoteichoic acid and peptidoglycan from Gram-positive bacteria [17, 42, 43].

In this study, we firstly propose that pHRODO bioparticles can be used to stimulate inflammation and to model impaired phagocytosis in DFU using a controlled ratio of diabetic fibroblasts to Mɸ, at defined oxygen and nutrient levels in vitro. Secondly, we hypothesize that phagocytic activity can be used as a new wound healing biomarker for drug screening purposes, given its critical role in determining the transit from inflammation to tissue proliferation during wound healing [10]. In neurodegenerative diseases such as Alzheimer’s disease, decline in the clearance of dying neurons and protein aggregates by microglia allows waste to accumulate, leading to microglial pro‐inflammatory activation in response to amyloid [44]. Interestingly, recent studies have reported stimulating phagocytosis and efferocytosis as promising therapeutic targets for treating neurodegenerative diseases [34, 45, 46]. Hence, given the common ground of defective phagocytosis between DFU and neurodegenerative diseases, drug re-purposing could be considered using the developed in vitro DFU model to identify drugs capable of stimulating phagocytosis on a high-throughput scale.

The present study confirmed that pHRODO *E. coli* and *S. aureus* bioparticles are effective at stimulating inflammation, and can be seamlessly used as proinflammatory stimuli—coupled with their established application as phagocytic cargos. Secondly, fibroblasts influence the phagocytic function of Mɸ, encouraging the use of the direct co-culture approach in DFU modelling whilst mimicking the dermis more closely. The human dermis is populated by fibroblasts and Mɸ [47]. The established in vitro DFU model demonstrated significant inflammation and reduced phagocytic activity towards pHRODO *S. aureus* bioparticles compared with the in vitro healthy wound model at a 1:4 ratio of fibroblasts to THP-1-derived Mɸ under hypoxia (2% oxygen) and reduced nutrient level (2% fetal bovine serum (FBS)). Lastly, significant stimulation of phagocytosis in the in vitro DFU model was observed following exposure to Bay 11–7085.

The reported approach of modelling DFU in vitro can be adopted for primary screening of drugs that could stimulate phagocytosis for DFU treatment.

## Materials and methods

### Cell culture

Sources of human fibroblasts included purchased adult dermal fibroblasts from healthy (AX3027; Lots #3027407 and #30272609) and type 2 diabetic individuals (AX3040; Lots #DDFM092518B and #DDFN100418A) from AXOL Bioscience Ltd. Upon receipt, the primary dermal fibroblasts were firstly grown using Fibroblast Plating & Growth medium plus Growth Factors (AX3045, Lot #10776, AXOL Bioscience Ltd.).

High glucose (HG) Dulbecco’s Modified Eagle’s Medium (DMEM) (D6429, Sigma Aldrich) was the base medium used for expanding fibroblasts. The fibroblasts were grown in complete HG DMEM (cHG-DMEM): HG-DMEM was supplemented with 10% FBS and 1% penicillin/streptomycin (P/S). The primary fibroblasts were seeded at 10,000 to 14,000 cells per cm^2^ and grown under typical culture conditions (37 °C, 5% CO_2_). The culture medium was changed every 3 days. At 80–90% confluence, fibroblasts were detached using trypsin–EDTA (5 min at 37 °C), neutralized using cHG-DMEM, centrifuged at 200 g for 5 min and resuspended in fresh cHG-DMEM.

THP-1, round suspension cells (TIB-202, Lot #70043382, American Type Culture Collection (ATCC)), were selected as human Mɸ source. This cell line was established from the peripheral blood of a 1-year old male with acute leukemia. THP-1 share common features with primary Mɸ including phagocytosis [48]. Furthermore, given the homogenous genetic background of THP-1, variability in cell phenotype is reduced; THP-1 can also be used for gene transfection studies.

Roswell Park Memorial Institute 1640 medium (R8758, Merck), supplemented with 10% FBS and 1% P/S, was used to cultivate THP-1 - abbreviated hereafter as “cRPMI”.

### Direct co-culture based wound model

THP-1 monocytes were differentiated into Mɸ using 10 ng/ml phorbol 12-myristate 13-acetate (PMA) in cRPMI for 24 h directly in the 96-well plate (SARSTEDT) used for the co-culture wound set-up (D0). Mɸ differentiation was followed by a 24 h resting period in cRPMI (D1). A previous work has shown that 10 ng/ml PMA treatment for 24 h followed by a 24 h rest time post-differentiation using cRPMI favoured significant cell adherence and viability of THP-1-derived Mɸ [49]. Then, fibroblasts (diabetic and healthy) were directly co-cultured with the rested THP-1-derived Mɸ in co-culture medium (CCM, i.e., 1:1 ratio of cHG-DMEM to cRPMI) for 24 h (D2). The pHRODO bioparticles were used as pro-inflammatory stimuli and phagocytic cargos on D3. Whereby hypoxic incubation was necessary, 37 °C and 2% oxygen settings were used from D0 to D3.

The pHRODO bioparticles used in this study were purchased from Thermo Fisher Scientific, namely: green *S. aureus* (Wood strain without protein A) with 509/533 nm excitation/emission λ (P35367), red *E. coli* (K-12 strain) with 560/585 nm excitation/emission λ (P35361) and deep red *E. coli* (K-12 strain) with 640/655 nm excitation/emission λ (P35361).

### Preparation of pHRODO bioparticles

Final concentrations of 1 mg/ml stock solutions were prepared by resuspending the pHRODO bioparticles in 2 ml of pre-warmed CCM, vortexed at 1300 rpm for 4 min, and stored at 4 °C for weeks, protected from light.

Upon adding pHRODO bioparticles (at the working concentrations) to the direct co-cultures, the 96-well plates were protected from light using aluminum foil and incubated for 4 h at the desired culture conditions.

### High content imaging and quantification

#### PerkinElmer Operetta High Content Imaging system

For imaging set-up, the PerkinElmer Operetta High Content Imaging system and the Harmony software were used. The µ-plate 96-well square plates (IBIDI, Catalogue #89,626) were used with the Operetta imaging system. The 20 × long working distance objective with a corresponding field of view of 675 × 509 µm was used—being the lowest magnification lens present. The “Non-Confocal” feature was selected and the lamp strength for excitation of fluorescent stains was specified to “50%.” The appropriate channels, based on the excitation and emission ranges of the used pHRODO bioparticles, were selected.

The most ideal exposure settings were set using a well containing a positive control by varying power and exposure time. The most focused plane within the same well was identified by setting up a stack in the “Layout Selection” tab—starting with a bottom plane of −10 µm up to 20 planes, using increments of 2.1 µm. The resulting stack was browsed to identify the plane with highly resolved emission of the pHRODO bioparticles, and the corresponding height value was manually transferred to the “Channel Selection” tab. After imaging the wells of interest, the “Evaluation” tab was used to obtain sets of images of interest.

#### SparkCyto Cell Imager system

Phagocytosis within the set-up direct co-cultures was quantified by measuring intracellular fluorescence emitted by engulfed pHRODO bioparticles at the corresponding emission wavelength(s) using the SparkCyto Cell Imager system (TECAN), operated via the SparkControl™ software. This system is equipped with multimode detection and fluorescence imaging features. It presents a highly advanced imaging module, three user-defined objectives, four fluorescence channels, light emitting diode-based illumination and autofocusing system; it also offers reproducibility and standardized procedures. Successful bioparticle uptake was imaged using high-definition phase contrast combined with fluorescent signals derived from the ingested pHRODO bioparticles within the acidic phagosomes of phagocytosing THP-1 derived Mɸ.

The 4× objective lens was used to enable imaging of the total fluorescence from most area of each well of the 96-well plate. This approach was to minimize data variability amongst imaged wells given its time effectiveness and could result useful for high-throughput drug screening applications.

There was no need for image segmentation due the significant fluorescence intensity from ingested pHRODO bioparticles over the background noise. The resulting fluorescence intensity data were cleaned using Microsoft Excel and reported as relative phagocytic activity units on plotted graphs using GraphPad Prism 10.

### ELISA

Supernatants used for ELISA were collected at defined timepoints and stored at − 20 or − 80 °C prior to quantifying TNF-α and MCP-1 levels in the set-up direct co-cultures. Supernatants from fibroblast monocultures were used to account for changes due to the pro-inflammatory stimulation effect on fibroblasts solely.

DuoSet ELISA kits for human MCP-1 (Catalogue #DY279-05) and TNF-α (Catalogue #DY210-05) detection were used along with the DuoSet Ancillary Reagent kit 2 (Catalogue #DY008) from R&D Systems. Each DuoSet kit contained human capture antibody, human detection antibody, human standard and streptavidin-horse radish peroxidase. The working dilutions of 1:50 and 1:100 were used for quantifying MCP-1 and TNF-α levels in collected supernatants from the direct co-cultures according to manufacturer’s operating procedures.

### Metabolic activity following drug treatment 

Firstly, pHRODO bioparticles and Bay 11-7085 (B5681, Merck) were co-delivered to the direct co-culture-based wound models for 4 h. Then, the resulting phagocytic activity was measured using the SparkCyto Cell Imager system. To identify non-cytotoxic concentrations of Bay 11-7085 that stimulate phagocytosis, metabolic activity was measured using the alamarBlue (AB) reagent assay (Thermo Fisher Scientific).

A 10% AB solution was prepared using CCM and added to the in vitro wound models (100 μL/well). The 96-well plate housing the direct co-culture-based wound models was protected from light using aluminum foil and incubated under normal culture conditions for 75 min. The resulting fluorescence was read using the Varioskan Flash plate reader (Thermo Fisher Scientific) at an excitation λ of 560 nm and an emission λ of 590 nm. 

### Statistical analysis

Results were confirmed by repeating experiments twice on separate occasions, where feasible. For clarity, data shown in each plotted graph were taken from single experiments. Data were presented as the mean of 3 technical replicates ± standard deviation (SD), unless stated otherwise in the figure legends.

Statistical analysis using one-way analysis of variance (ANOVA) and graphing were conducted using GraphPad Prism 10 software. Differences between groups were considered significant at *p* < 0.05 using Tukey’s and Dunnett’s where applicable. For statistical significance, *p* values in individual graphs were represented as follows: 0.1234 (ns^1^), 0.0332 (*), 0.0021 (**), 0.0002 (***), and 0.0001 (****).

## Results

### pHRODO bioparticles are capable of stimulating inflammation in vitro

Previous studies in diabetes-related complications have demonstrated that MCP-1 is secondary to TNF-α in type 2 diabetes and MCP-1 signalling attenuates TNF-α expression [50, 51]. Interestingly, the concurrent release of TNF-α and MCP-1 has been used to model inflammation in DFU in vitro in a previously reported direct co-culture-based DFU model using murine cell lines [17]. In fact, when TNF-α expression was silenced with anti-TNF-α lipidoid nanoparticles, both TNF-α and MCP-1 levels were significantly reduced both in vitro and in in vivo (using a double-wounded diabetic murine model, which showed increased wound healing rate).

Given the bacterial constituents of the chosen pH-sensitive bioparticles, it was expected that they can induce a pro-inflammatory response similarly to the commonly used pro-inflammatory stimulants. Such capability of pHRODO bioparticles has not been reported explicitly in the literature hitherto for in vitro disease modelling. Furthermore, both pHRODO *S. aureus* and *E. coli* bioparticles are patho-physiologically relevant in DFU modelling as both bacteria have been detected in diabetic foot infections [52].

Following skin tissue injury, the dermis—which is mainly populated by fibroblasts and Mɸ—becomes exposed.

Hence, using both cell types in in vitro wound modelling is justifiable. Four different direct co-culture ratios were used to test the inflammation-inducing ability of pHRODO bioparticles, namely: 100:0, 80:20, 50:50, and 20:80 fibroblasts to THP-1-derived Mɸ. Till date and to the best of our knowledge, optimizing the direct co-culture ratio of fibroblasts to Mɸ in modelling DFU in vitro has not been reported. The rationale behind the selected direct co-culture ratios was that, during inflammation, there are temporary increases of specific cell populations especially in functional and organizational circuits involving fibroblasts and Mɸ [53, 54]. In fact, previous murine and human studies reported that around 80% of cells present in diabetic wounds are Mɸ with a pro-inflammatory (M1) phenotype, coupled with impaired transition to an anti-inflammatory (M2) phenotype, hence impeding progression of tissue repair [14]. The highly pre-dominant M1 Mɸ presence results in high proinflammatory mediator and matrix metalloproteinase levels, ECM degradation, and reduced keratinocyte and fibroblast proliferation and migration [13].

A preliminary timepoint study was conducted using LPS as a pro-inflammatory stimulant in the different direct co-culture ratios; 4-h exposure to LPS was identified as the optimum timepoint for significant and concurrent detection of TNF-α and MCP-1 (Supplementary Fig. 2). Furthermore, the non-cytotoxicity of the tested working concentration range of pHRODO bioparticles (0–800 µg/ml) following 4-h exposure was confirmed (Supplementary Fig. 3).

**Fig. 1:**
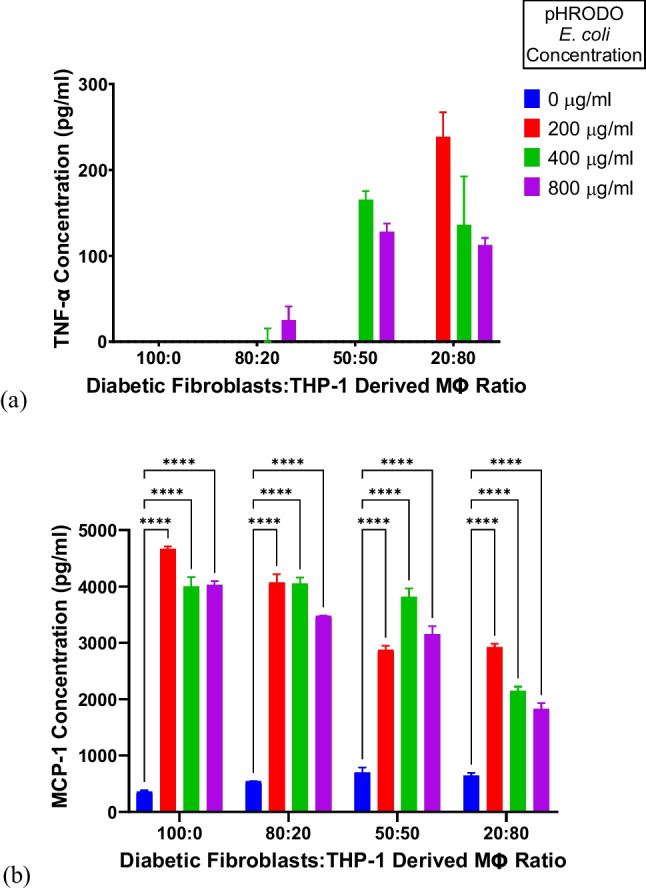
Pro-inflammatory cytokine release following pHRODO bioparticle exposure. (**a**) TNF-α and (**b**) MCP-1 release by different co-culture ratios of diabetic dermal fibroblasts to THP-1-derived Mɸ following 4-h exposure to different concentrations of pHRODO deep red *E. coli* bioparticles using ELISA. Data points are represented as mean values ± SD (*n=2*). Statistical significance (*p* value) = 0.1234 (ns), 0.0332 (*), 0.0021 (**), 0.0002 (***), and 0.0001 (****)

**Fig. 2:**
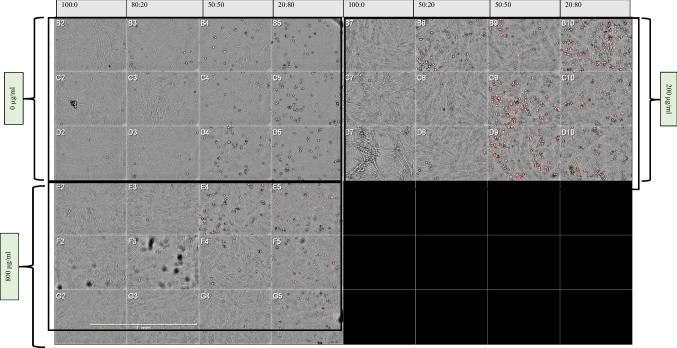
Imaging of the uptake of pHRODO deep red *E. coli* bioparticles using the Operetta system. Representative micrographs for the different co-culture ratios of diabetic fibroblasts to THP-1-derived Mϕ with different pHRODO deep red *E. coli* bioparticle concentrations (20x magnification). One representative field per well in triplicate was captured. The black cells contained no cells. Spindle-shaped cells represent fibroblasts; round-shaped cells represent THP-1-derived Mɸ. Scale bar shown as 1 mm

To confirm the proinflammatory ability of pHRODO bioparticles, the chosen direct co-culture ratios were exposed to different concentrations of pHRODO deep red *E. coli* bioparticles (0, 200, 400, and 800 µg/ml) for 4 h. Noticeably, fibroblasts were the key producers of MCP-1, which was significantly produced upon exposure to all pHRODO *E. coli* concentrations. Furthermore, a concentration-dependent decrease in MCP-1 production was observed with increasing presence of THP-1 derived Mɸ. After 4 h of pHRODO deep red *E. coli* bioparticle exposure, the 20:80 ratio of diabetic dermal fibroblasts to THP-1-derived Mɸ released significantly high levels of TNF-α and MCP-1 concurrently compared with the other direct co-culture groups at all tested pHRODO concentrations (Fig. 1a–b). Particularly, there was evidently most uptake of pHRODO deep red *E. coli* bioparticles at 200 µg/ml. Representative images of the successful uptake of 200 and 800 µg/ml pHRODO deep red *E. coli* bioparticles are shown in Fig. 2. Therefore, the 20:80 ratio of fibroblasts to THP-1-derived Mɸ exposed to 200 µg/ml pHRODO bioparticles was selected to model inflammation in DFU.

**Fig. 3:**
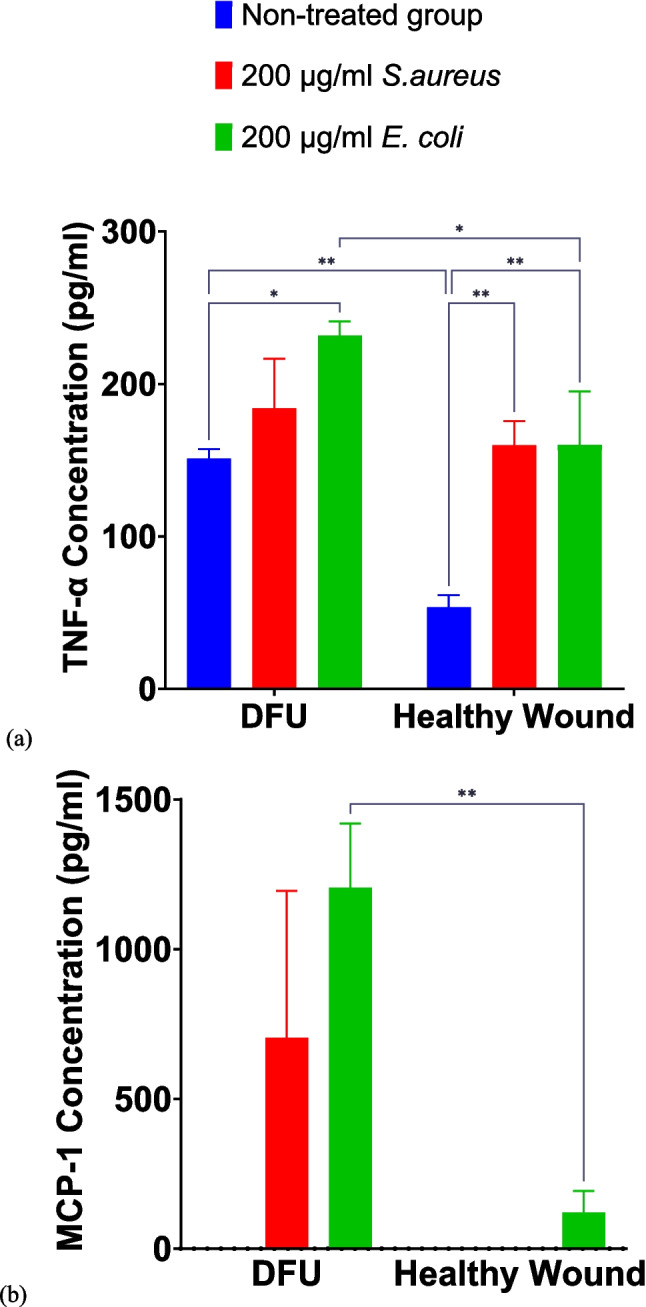
Pro-inflammatory cytokine release following pHRODO bioparticle exposure in the 20:80 fibroblasts to THP-1-derived Mɸ ratio (**a**) TNF-α and (**b**) MCP-1 release after four-hour exposure to different pHRODO bioparticles under normoxia using CCM containing 10% FBS for bioparticle preparation. Data points are represented as mean values ± SD (*n* = 2). Statistical significance (*p* value) = 0.1234 (ns), 0.0332 (*), 0.0021 (**), 0.0002 (***), and 0.0001 (****)

Both pHRODO *S. aureus* and *E. coli* bioparticles in CCM containing 10% FBS stimulated significantly higher levels of TNF-α and MCP-1 in the 20:80 ratio of diabetic dermal fibroblasts to THP-1-derived Mɸ in comparison with the healthy wound counterpart under normoxia (Fig. 3). A similar effect was observed under different combinations of oxygen levels and FBS concentrations (Supplementary Figs. 4–6).

Based on these data, the in vitro DFU model exhibited different responses in the soluble factor secretion into the surrounding microenvironment as a function of used “baits” for phagocytosis.

### The addition of LPS does not stimulate the phagocytosis of pHRODO *S. aureus* and *E. coli* bioparticles in the in vitro wound models

Previous literature has reported that LPS treatment resulted in phagosome maturation, upregulated membrane-trafficking regulators and lysosomal enzymes, bactericidal activity, and improved phagocytosis [55, 56]. Hence, in both direct co-culture-based DFU and healthy wound models, the pHRODO *E. coli* and *S. aureus* bioparticles were separately co-delivered with different LPS concentrations (0.5–5 μg/ml) to investigate the effect of LPS on the resulting phagocytic activity.

**Fig. 4:**
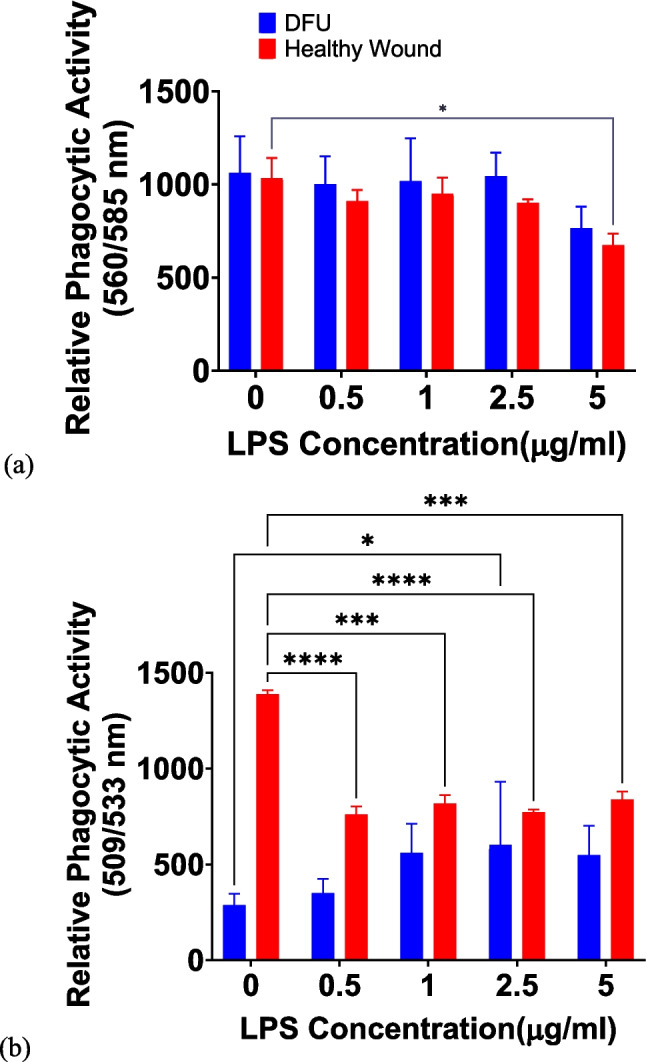
The effect of LPS on the uptake of pHRODO bioparticles. The resulting phagocytic activity following exposure to (**a**) pHRODO red *E. coli* bioparticles and (**b**) pHRODO green *S. aureus* bioparticles for 4 h in both the DFU and healthy wound direct co-culture based models. Data points are represented as mean values ± SD (*n* = 3). Statistical significance (*p* value) = 0.1234 (ns), 0.0332 (*), 0.0021 (**), 0.0002 (***), and 0.0001 (****)

Irrespective of LPS concentration, the uptake of pHRODO red *E. coli* bioparticles did not differ between the in vitro DFU and healthy wound models (Fig. 4a). The exception to this observation was for the in vitro healthy wound model treated with 5 μg/ml LPS, whereby a significant reduction in phagocytosis was noticed relative to the untreated group.

On the other hand, the uptake of pHRODO green *S. aureus* bioparticles by the in vitro healthy wound model was significantly reduced in the presence of LPS compared with the untreated group, whereas there was no observable increase in phagocytic activity in the in vitro DFU model except for 2.5 μg/ml LPS (where a slightly significant increase in phagocytic activity was observed) (Fig. 4b).

Hence, LPS supplementation had no synergistic effect on the phagocytic activity of the direct co-culture based wound models towards both pHRODO *E. coli* and *S. aureus* bioparticles in vitro.

### The uptake of pHRODO *E. coli* bioparticles does not differ between the in vitro DFU and healthy wound models

Peripheral arterial disease (PAD) is amongst the triad leading to DFU development [56, 57]. PAD—characterized by occluded lower extremity arteries, pain and functional impairments—results in tissue ischaemia in the lower limbs. Ischaemia is characterized by reduced blood flow, and hence decreased oxygen and nutrient supply, to the affected lower limb [58]. In this study, different combinations of oxygen and nutrient levels were assessed to identify DFU-like in vitro parameters.

To simulate pathological and physiological nutrient levels in wound healing, two different FBS concentrations were used in preparing the pHRODO bioparticle solutions: 2% and 10%, respectively. Similarly, to mimic pathological and physiological oxygen levels, hypoxia (2% oxygen) and normoxia (20% oxygen) were used for setting up the in vitro wound models, respectively.

**Fig. 5:**
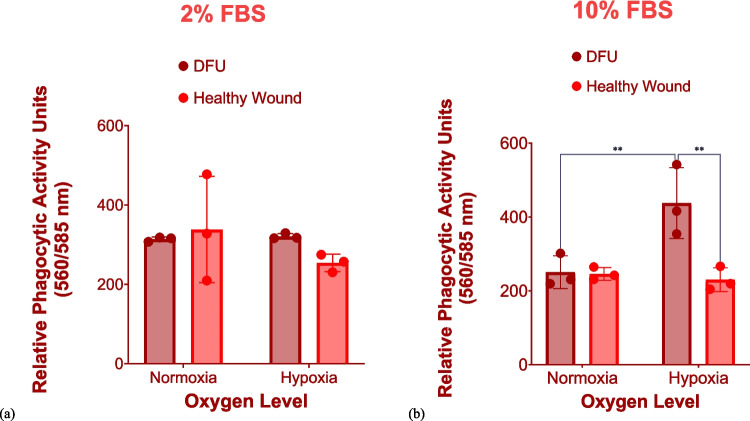
Phagocytosis of pHRODO red *E. coli* bioparticles in both in vitro wound models. Both graphs show the relative phagocytic activity towards pHRODO red *E. coli* bioparticles by the in vitro DFU and healthy wound models after 4 h exposure using (**a**) 2% FBS and (**b**) 10% FBS under different oxygen levels. Data points are represented as mean values ± SD (*n* = 3). Statistical significance (*p* value) = 0.1234 (ns), 0.0332 (*), 0.0021 (**), 0.0002 (***), and 0.0001 (****)

At the low nutrient level (i.e., 2% FBS), the in vitro DFU model demonstrated comparable phagocytic activity towards 200 μg/ml pHRODO red *E. coli* bioparticles to the in vitro healthy wound model, irrespective of oxygen level (Fig. 5a). Likewise, comparable phagocytic activity was observed between both in vitro wound models exposed to *E*. *coli* bioparticles under normoxia, irrespective of FBS concentration. However, at the high nutrient level (i.e., 10% FBS), significantly higher uptake of pHRODO *E. coli* bioparticles was observed in the in vitro DFU model under hypoxia compared with normoxia (Fig. 5b). Additionally, using the high nutrient level (i.e., 10% FBS) under hypoxia, the in vitro DFU model significantly phagocytosed more *E. coli* bioparticles compared with the in vitro healthy wound counterpart (Fig. 5b).

Representative images of the uptake of pHRODO red *E. coli* bioparticles under some of the considered in vitro microenvironmental parameters are shown in Supplementary Figs. 7 and 8.

Hence, based on the findings, the sole use of pHRODO *E. coli* bioparticles for developing the in vitro DFU model (characterized by both significant inflammation and reduced phagocytic activity) was deemed non-ideal.

### The in vitro DFU model had the significantly least phagocytic activity towards pHRODO green *S. aureus* bioparticles compared with the in vitro healthy wound model under hypoxia and the low nutrient level

**Fig. 6:**
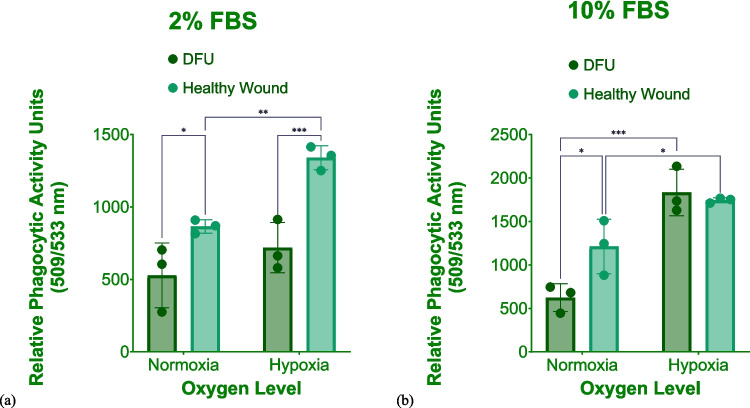
Phagocytosis of pHRODO green *S. aureus* bioparticles in both in vitro wound models. Both graphs show the relative phagocytic activity of the in vitro DFU and healthy wound models towards pHRODO green *S. aureus* bioparticles after 4 h exposure using (**a**) 2% FBS and (**b**) 10% FBS under different oxygen levels. Statistical significance (*p* value) = 0.1234 (ns), 0.0332 (*), 0.0021 (**), 0.0002 (***), and 0.0001 (****)

Under the low nutrient level (i.e., 2% FBS), the in vitro DFU model demonstrated significantly reduced phagocytic activity towards pHRODO green *S. aureus* bioparticles compared with the in vitro healthy wound model under both oxygen levels (Fig. 6a and b). Furthermore, under normoxia and the high nutrient level (i.e., 10% FBS), the in vitro DFU model still demonstrated significant reduction in phagocytic activity compared with the in vitro healthy wound model.

Based on the obtained data, hypoxia significantly stimulated the uptake of *S. aureus* bioparticles in the in vitro healthy wound model. Particularly, the in vitro DFU model had the significantly least phagocytic activity towards *S. aureus* bioparticles under hypoxia and at the relatively lower nutrient level compared with the in vitro healthy wound model. This finding correlates with the pathological traits of DFU in in vivo: coupled with hyperglycaemia, tissue ischaemia and hypoxia, due to PAD, interrupt the progression towards tissue repair. Reduced phagocytic activity in the in vitro DFU model compared with the in vitro healthy wound model was also observed under normoxia with both FBS concentrations.

**Fig. 7:**
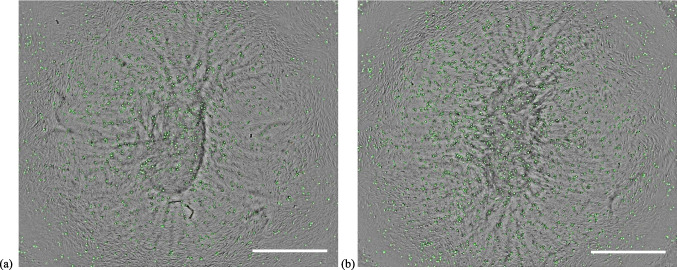
Phagocytosis of pHRODO green *S. aureus* bioparticles in both in vitro wound models. The uptake of pHRODO green *S. aureus* bioparticles by (**a**) in vitro DFU model and (**b**) the in vitro healthy wound model under hypoxia using 2% FBS (4× magnification). Scale bar shown is 100 µm

Representative images of phagocytosed pHRODO green *S. aureus* bioparticles in CCM supplemented with 2% FBS under hypoxia in both in vitro wound models are shown on Fig. 7. Representative images of the uptake of *S. aureus* bioparticles in CCM containing 10% FBS under both oxygen levels are provided in Supplementary Figs. 9 and 10.

Hence, pHRODO green *S. aureus* bioparticles in CCM containing 2% FBS coupled with hypoxic incubation could be proposed as both proinflammatory stimuli and phagocytic cargos for modelling both significant inflammation and reduced phagocytic capability of DFU in vitro.

### Increased phagocytic activity observed in the direct co-culture-based wound models compared with Mɸ only

Fibroblast dysfunction in DFU results in secondary complications such as reduced re-epithelialization and angiogenesis [58]. Given the plasticity of Mɸ, the negative impact of the dysfunctional microenvironment of DFU on its phagocytic function represented by the incorporation of diabetic fibroblasts in the direct co-culture-based wound model set-up can be hypothesized.

Hence, the phagocytic activity of both in vitro wound models was compared with that of THP-1-derived Mɸ only. A 1:1 ratio of pHRODO green *S. aureus* and pHRODO red *E. coli* bioparticles was employed to induce inflammation and quantify phagocytic activity after 4 h.

**Fig. 8:**
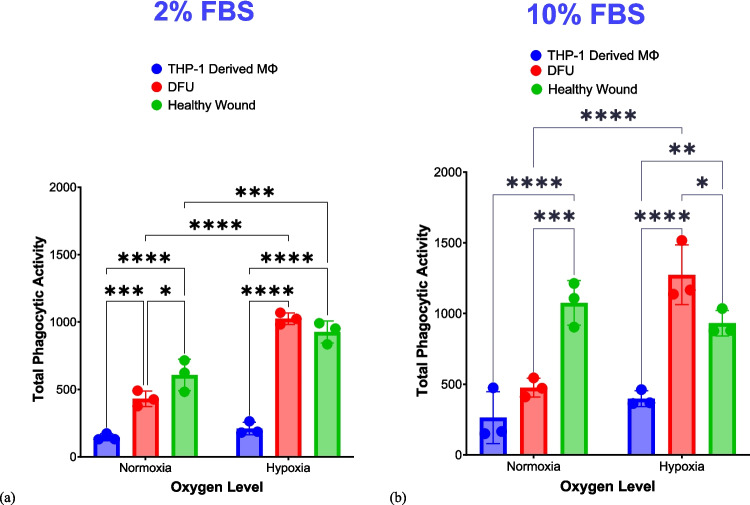
The immunomodulatory effect of fibroblasts on the phagocytic activity of the in vitro wound models towards pHRODO *E. coli* and *S. aureus* bioparticles. Phagocytic activity towards 1:1 ratio of pHRODO *S. aureus*:*E. coli* bioparticles after 4-h exposure by diabetic and healthy wound direct co-culture models using (**a**) 2% FBS and (**b**) 10% FBS at different oxygen levels. Data points are represented as mean values ± SD (*n* = 3). Statistical significance (*p* value) = 0.1234 (ns), 0.0332 (*), 0.0021 (**), 0.0002 (***), and 0.0001 (****)

A significant increase in the phagocytic activity towards pHRODO *E. coli* and *S. aureus* bioparticles (1:1 ratio) was observed in both in vitro wound models compared with that of the THP-1-derived Mϕ only group, irrespective of FBS concentration and oxygen level (Fig. 8a–b). This finding is representative of the overall immunomodulatory effect provided by supporting cells such as fibroblasts in achieving successful tissue repair.

**Fig. 9:**
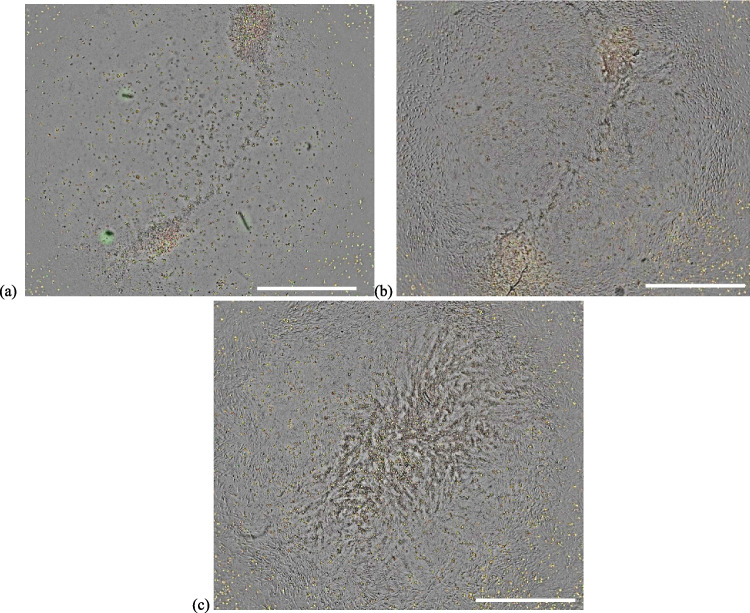
The uptake of pHRODO red *E. coli* and green *S. aureus* bioparticles in both in vitro wound models compared with THP-1-derived Mɸ only. The phagocytic activity of (**a**) THP-1 Mɸ only, (**b**) the in vitro DFU and (**c**) the in vitro healthy wound models towards 1:1 ratio of pHRODO green *S. aureus*:red *E. coli* bioparticles under normoxia at 10% FBS (4× magnification). The successful uptake of both pHRODO green *S. aureus* and red *E. coli* bioparticles within a phagocyte gives a yellow colouration in the resulting images. Scale bar shown is 100 μm

The phagocytic activity of the in vitro healthy wound model increased by twofold with increasing FBS concentration under normoxia. This latter result possibly shows the contributory effect of increased concentration of opsonins to phagocytosis. Opsonins are present in FBS (e.g., antibodies and complement proteins) and they tag foreign pathogens and render them more recognizable for clearance by phagocytes [60, 61]. Particularly, under normoxia, the phagocytic activity of the in vitro DFU model towards the 1:1 ratio of pHRODO green *S. aureus* and red *E. coli* bioparticles was significantly reduced compared with the in vitro healthy wound counterpart, irrespective of nutrient level. Representative images of phagocytosed pHRODO green *S. aureus* and red *E. coli* bioparticles in CCM supplemented with 10% FBS under normoxia in both in vitro wound models compared with THP-1-derived Mɸ only are shown on Fig. 9.

In the present context, the differing factor in both in vitro wound models, resulting in differences in phagocytic activity, was the fibroblast source: THP-1-derived Mɸ were directly co-cultured with diabetic fibroblasts in the in vitro DFU model, whereas fibroblasts from non-diabetic individuals were used for the in vitro healthy wound model. Overall, these findings suggest how the fibroblast source significantly influences wound healing dynamics.

### Bay11-7085 stimulated phagocytosis in the in vitro DFU model

Amongst the combined oxygen and FBS concentration parameters that resulted in significant reduction in phagocytic activity towards pHRODO *S. aureus* bioparticles, the chosen in vitro DFU model for drug testing was set up using pHRODO bioparticles in CCM containing 10% FBS under normoxia. This choice as the in vitro DFU model for drug testing, rather than 2% FBS under hypoxia, served to simulate the use of pro-healing agents as adjuvant treatments, specifically topical oxygen therapy (mimicked by normoxia) and growth factor therapy (using pHRODO *S. aureus* bioparticles prepared in CCM supplemented with 10% FBS).

To verify the usefulness of the in vitro DFU model, the kinase Bay 11–7085, an inhibitor of nuclear factor-kappa B (NF-κB) activation and inhibitory κB-α(IκB-α) phosphorylation, was used as previous in vitro studies showed its stimulatory effect on phagocytosis [41, 62]. NF-κB is a family of inducible transcription factors, which regulates a large array of genes involved in different processes of the immune and inflammatory responses [55]. Bay 11-7085 inhibits the dissociation of IκB-α from NF-κB, hence blocking TNF-α-induced IκB-α phosphorylation and, in turn, the transcription of pro-inflammatory mediators.

**Fig. 10:**
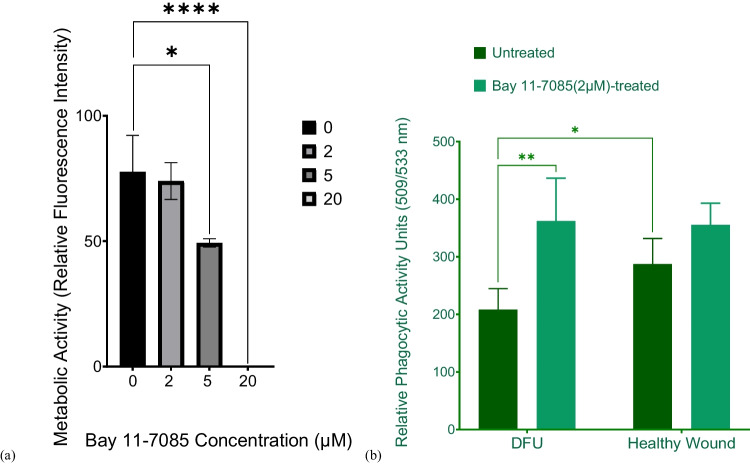
The effect of Bay 11-7085 on the viability and phagocytic activity of the in vitro wound models. (**a**) The viability of the in vitro DFU model in the presence of different Bay11-7085 concentrations and pHRODO green S. aureus bioparticles and (**b**) the phagocytic activity towards pHRODO green S. aureus bioparticles of untreated and Bay 11-7085-treated groups of the direct co-culture based wound models. Data points are represented as mean values ± SD (n = 4). Statistical significance (*p* value) = 0.1234 (ns), 0.0332 (*), 0.0021 (**), 0.0002 (***), and 0.0001 (****)

Firstly, the effect of different concentrations of Bay 11–7085 on the metabolic activity of the in vitro DFU model was tested in the presence of pHRODO green *S. aureus* bioparticles. Significant reduction in metabolic activity was observed at all considered Bay 11-7085 concentrations except for 2 µM (Fig. 10a).

Therefore, 2 µM Bay 11-7085 was co-delivered with pHRODO green *S. aureus* bioparticles in both in vitro wound models. Interestingly, there was a significant increase in the phagocytic activity in the in vitro DFU model towards pHRODO green *S. aureus* bioparticles in the presence of Bay 11–7085 compared with the healthy wound counterpart (Fig. 10b). On the other hand, the in vitro healthy wound model showed no difference in phagocytic activity following Bay 11-7085 treatment.

## Discussion

In this work, pHRODO bioparticles were applied to model inflammation and reduced phagocytosis in DFU in vitro for the first time to the best of our knowledge*.*

The pHRODO bioparticles were confirmed to stimulate inflammation in the direct co-culture-based wound models (a 1:4 ratio of fibroblasts to THP-1 derived Mɸ)—via TNF-α and MCP-1 quantification. The observed proinflammatory capacity of pHRODO bioparticles is supported by previous studies. Firstly, high transcript levels of TNF-α, CCL4, CCL5 and PTGS2 were observed following one- or four-hour exposure to pHRODO *E. coli* bioparticles in murine Mɸ [102]. Secondly, endocrine-resident Mɸ isolated from murine pancreas, upon 4-h exposure to pHRODO red *E. coli* bioparticles, secreted detectible levels of TNF-α, IL-1β and IL-6 in vitro [30].

The addition of LPS reduced the uptake of pHRODO *E. coli* and *S. aureus* bioparticles in both in vitro DFU and healthy wound models. LPS acts primarily on Toll-like Receptor 4 (TLR-4), whereas the multiple pathogen associated molecular patterns (PAMP) on the chemically killed whole *E.* *coli*/*S. aureus* conjugated to the pHRODO dye interact with distinct pattern recognition receptors on THP-1-derived Mɸ to upregulate receptors involved in phagocytosis and inflammation [63]. The inhibitory effect of LPS on pHRODO bioparticle uptake can be justified as follows: the binding and uptake rate of LPS could be faster than that of pHRODO bioparticles, resulting in endotoxin tolerance. In fact, Kapellos et al. found that, after 16-h LPS pretreatment, the uptake of pHRODO green *E. coli* bioparticles by bone marrow-derived Mϕ was significantly inhibited in a LPS concentration-dependent manner [39]. Similarly, another study reported decreased uptake of pHRODO-labelled myelin by bone marrow-derived Mɸ following LPS pretreatment [62]. Collectively, the reducing effect of LPS on pHRODO bioparticle uptake could be due to endotoxin tolerance, whereby prior exposure to even low concentrations of endotoxin (e.g., LPS) causes cells to become transiently unresponsive to further endotoxin challenges such as pHRODO bioparticles. Hence, pHRODO bioparticles solely can be used to stimulate inflammation effectively, coupled with their use as phagocytic cargos.

Amongst the combinations of different types of pHRODO bioparticles, oxygen levels and FBS concentrations tested, the pHRODO green *S. aureus* bioparticles successfully induced inflammation and were least phagocytosed by the in vitro DFU model compared with the healthy wound counterpart when delivered in CCM containing 2% FBS under hypoxia. This key finding is justified by previous works.

Firstly, both *S. aureus* and the *Streptococcus genera* have been predominantly detected in DFU with mild or moderate infection [64]. Additionally, some in in vivo works found that, although leukocytes are significantly recruited in diabetic wounds, prolonged infection occurs due to defective phagocytic ability following *S. aureus* challenge [65, 66]. Secondly, the identified low oxygen (2% oxygen) and nutrient (2% FBS) levels associated with reduced phagocytic activity in the in vitro DFU model correlate with DFU pathophysiology in in vivo*—*characterized by the presence of PAD, which is manifested as reduced blood-flow (hence, reduced oxygen and nutrient supply) to the affected lower limb [57, 67]. Interestingly, a clinical study reported how ischaemic DFU cases had the worst healing rate compared with both neuropathic and neuro-ischaemic DFU cases [68].

Under hypoxia, the transcriptional regulator hypoxia inducible factor-1 alpha (HIF-1α) stably translocates into the nucleus and dimerizes with HIF-1β to form the HIF-1 heterodimer, resulting in the transcription of target genes to ensure cell survival and function under oxygen-deprived conditions [69, 70]. In DFU, however, due to hyperglycaemia and oxidative stress, reduced HIF-1α stability ensues, resulting in reduced neovascularization and, eventually, poor tissue repair [71, 72]. Overall, hypoxia develops in DFU due to imbalances between oxygen demand and supply. Hence, the significantly reduced phagocytic activity towards pHRODO green *S. aureus* bioparticles in the in vitro DFU model under hypoxia and using 2% FBS compared with the healthy wound counterpart could be partly due to HIF-1α inactivation. Interestingly, some studies till date have shown that the deletion of *Hif-1a* gene within myeloid cells is associated with impaired phagocytosis [73, 74]. For example, Anand et al. reported that hypoxia significantly increased phagocytosis rate and microbicidal activity in murine and human peritoneal Mɸ, and in mice exposed to systemic hypoxia. Particularly, phagocytosis was facilitated via HIF-1α-dependent expression. In fact, when HIF-1α was knocked down, phagocytic activity was negatively affected [73]. Similarly, in human volunteers, intermittent exercise-induced hypoxia resulted in increased phagocytosis and clearance of *E. coli* by neutrophils [75]. Remarkably, a study found that the *Hif-1a* transcriptional pathway in myeloid cells was significantly upregulated in murine bone marrow-derived Mɸ following exposure to either group A *Streptococcus* (GAS), *S. aureus*, *P. aeruginosa *or *Salmonella typhimurium* even under normoxic conditions (independent of hypoxia). Particularly, the expression of granule protease and cathelicidin was significantly reduced in HIF-1α–deficient neutrophils, which is associated with diminished bactericidal activity in vitro and inability to fight infection in in vivo [76].

Under normoxia at the high nutrient level (i.e., 10% FBS), the in vitro DFU model demonstrated significantly reduced uptake of *S. aureus* bioparticles compared with the in vitro healthy wound model. Chronic wounds such as DFU tend to present *S. aureus* as small aggregates near the wound surface, whereas *P. aeruginosa* resides in deeper tissues [77, 78]. Hence, the deficits in phagocytosis in DFU in vitro under normoxia, even at the high nutrient level, could be related to the established abundant presence of *S. aureus* in the foot of the diabetic population [79]. Furthermore, the in vitro DFU model ingested significantly less pHRODO *S. aureus* and *E. coli* bioparticles (1:1 ratio) compared with the in vitro healthy wound model under normoxia. These observations support the reduced phagocytic ability in diabetic individuals towards polymicrobial infections [57, 80].

Under hypoxia, both in vitro DFU and healthy wound models exhibited comparable phagocytic activity towards *S. aureus* bioparticles delivered in CCM containing 10% FBS (i.e., the high nutrient level). Wound healing is a catabolic process, justifying the need for increased nutrition [58]. However, diabetic individuals are prone to malnutrition due to metabolic changes such as hyperglycaemia and microalbuminuria, which could adversely affect phagocytosis during tissue repair [81]. The observed improvement in phagocytosis in the in vitro DFU model could be associated with the positive effect of increased nutrient supply, which possibly parallels with the promising efficacy of topical growth factor-based therapies used for DFU treatment [82]. Interestingly, a case–control study reported that, following short-term blood glucose optimization, the phagocytic activity of peripheral blood mononuclear cells (using pHRODO *E. coli* bioparticles) significantly increased in individuals with type 2 diabetes [83]. Additionally, serum albumin levels, used to assess nutritional status and liver function, were significantly lower in individuals with DFU that underwent an amputation compared with those that did not [84]. Hence, improving nutritional status in DFU could contribute to tissue repair, including supporting phagocytosis.

Multi-faceted immunomodulatory effects of fibroblasts on myeloid cells exist which regulate immune cell behavior, tissue repair and regeneration. Fibroblasts attract immune cells to the injured/infected tissue and interact with them to modulate their response via their inflammatory secretome such as chemokines, cytokines and interleukins. Upon interaction with immune cells, the activated fibroblasts produce further inflammatory mediators, creating a positive feedback loop. In DFU, specifically, fibroblast activity is impaired by overstimulation of the immune system due to the presence of LPS, advanced glycation end-products and high reactive oxygen species levels, resulting in a prolonged inflammatory phase. This latter is further prolonged by the activated Notch signaling, preventing the trans-differentiation of fibroblasts into myofibroblasts [59]. This pre-existing knowledge embodies the observed immunomodulatory effect of fibroblasts in both in vitro wound models, which resulted in an overall increase in phagocytic activity compared with the THP-1-derived Mɸ group only. Furthermore, the in vitro DFU model showed reduced phagocytic activity compared with the in vitro healthy wound model when challenged with 1:1 ratio of pHRODO *E. coli* and *S. aureus* bioparticles under normoxia irrespective of FBS concentration. This observation highlights the influence of fibroblast health status on the resulting Mɸ function and provides possible parameters to mimic defective phagocytosis towards polymicrobial infections in DFU in vitro. Interestingly, previous works have confirmed relatively higher phagocytosis of bacteria by Mɸ in the presence of fibroblasts. For example, direct co-cultures of Mɸ and oral fibroblasts from periodontal ligament significantly improved the phagocytosis of viable *P. gingivalis* compared to Mϕ only, coupled with reduced proinflammatory cytokine responses [28]. Furthermore, Maione et al. [22] found that full-thickness, excisional wounds in non-diabetic mice injected with hydrogel loaded with non-diabetic foot fibroblasts healed significantly faster than those treated with either diabetic foot fibroblasts or DFU-derived fibroblasts, between 2 and 5 days post-wounding. Though not investigated, the immunomodulatory effect of fibroblasts on Mϕ activity during the early phase of tissue repair, including phagocytosis, could be hypothesized.

Previously, inhibiting the NF-κB signalling pathway has been shown to stimulate phagocytosis in vitro. For example, Wu et al. reported that 2 μM Bay 11-7082 (a NF-κB inhibitor) completely restored the phagocytosis of pHRODO Green STP Ester-labelled myelin isolated from cerebral cortices of adult rat brains by RAW264.7 Mϕ in the presence of LPS [62]. Recently, Bay 11–7085 (up to 8 µg/ml) showed potent anti-microbial and biofilm activities towards *S. aureus* [85]*.* Bay 11–7085 demonstrated efficacy against the three phases of *S. aureus* biofilm maturation via inhibiting initial cell attachment, biofilm formation and maturation. Furthermore, Bay 11–7085 demonstrated stronger anti-fungal activity towards *Candida* species, even in mature biofilms with *S. aureus* in vitro. Bay 11–7085 was capable of penetrating through the surrounding matrix of biofilms, resulting in the direct killing of biofilm-residing bacteria and fungi, coupled with reduced virulence factors. Given the reported potent activity of Bay 11–7085 against *S. aureus*, *C. albicans*, and associated biofilms, it was hence recommended for topical wound management. Interestingly, heat-inactivated *S. aureus* (HISA) upregulated both NF-κB cascade gene expression and its phagocytosis by murine RAW 246.7 Mϕ [41]. However, upon the inhibition of the NF-κB signalling pathway, the phagocytosis of HISA was inhibited. Therefore, NF-κB activation was deemed necessary for HISA phagocytosis by Mϕ. This finding corroborates previous in in vivo studies where a robust yet controlled inflammation is essential for successful tissue repair [86]. Therefore, although a NF-κB inhibitor, Bay 11–7085 stimulated significant uptake of phRODO green *S. aureus* bioparticles in the in vitro DFU model compared with the in vitro healthy wound model, possibly due to using a relatively lower concentration. Collectively, though reportedly effective in both improving phagocytosis and bacterial/fungal killing, the cytotoxicity profile and mechanism of action of Bay 11–7085 warrant further investigation.

The major findings from this study are as follows. Firstly, the identification of parameters for modelling inflammation and impaired phagocytosis in DFU in vitro *– *consisting of a 1:4 ratio of diabetic fibroblasts to THP-1-derived Mɸ in direct co-culture exposed to 200 µg/ml pHRODO green *S. aureus* bioparticles in CCM containing 2% FBS for 4 h under hypoxia. Secondly, Bay 11–7085 resulted capable of stimulating phagocytosis in the reported in vitro DFU model, similarly to previous in vitro studies. The reported in vitro DFU model can be proposed for the primary screening of potential therapies that can stimulate phagocytosis for treating DFU.

### Limitations of the study and future considerations

#### Origin of cell types used

The developed direct co-culture-based DFU model used human diabetic dermal fibroblasts and THP-1-derived Mϕ. The differentiation of Mϕ from THP-1 was solely confirmed via adherence to tissue culture plastic following PMA treatment accompanied by some morphological changes. Though sufficient for confirming monocyte differentiation into Mϕ, it will be interesting to confirm using flow cytometry, immunofluorescence (via the detection of distinct surface markers on THP-1-differentiated Mϕ such as CD14, CD35, CD55 and CD59) or other appropriate techniques [87].

To ensure data robustness and avoid phenotypic drift, low passages of primary dermal diabetic fibroblasts were used in the present work and are strongly recommended. Although the use of THP-1 cell line favours reproducibility, however, it does not reflect the phagocytic capability of diabetic Mɸ. Hence, to achieve physio-pathological relevance in future works, Mϕ located within active DFU tissues can be isolated from debrided tissues or monocytes isolated from peripheral blood from individuals with DFU followed by differentiation into Mϕ can be used for setting up the in vitro DFU model, given the intrinsic functional alterations that could be peculiar to DFU-derived Mϕ. Based on data derived from murine and human studies, it can be collectively concluded that diabetic Mϕ are hyper-responsive to proinflammatory stimulation, resulting in significant proinflammatory cytokine release, which impedes M2 transition and protracts the inflammatory phase [13]. Additionally, Mϕ exposed to high glucose in vitro presented reduced phagocytic activity, coupled with decreased nitric oxide secretion [88, 89].

Similarly, it is ideal to use fibroblasts isolated from debrided DFU tissues or discarded skin specimens from routine surgical procedures such as amputation, bunionectomy and arthroplasty, involving individuals with a DFU [22, 24, 90, 91]. Published works by Maione et al. reported the altered effects of DFU-derived fibroblasts compared with both diabetic and non-diabetic fibroblasts on some wound healing processes [22, 90]. For example, in vitro 3D human skin equivalent (HSE) models containing fibroblasts from diabetic patients without DFU achieved re-epithelialization, similarly to those containing non-diabetic fibroblasts—as opposed to HSE containing DFU-derived fibroblasts which were less supportive of keratinocyte migration. The HSE contained keratinocytes derived from non-diabetic skin tissue. Additionally, endothelial sprouting was significantly reduced in the presence of DFU-derived fibroblasts than in the presence of foot-derived fibroblasts from either site-matched diabetic or non-diabetic individuals.

Lastly, DFU-derived fibroblasts and Mɸ cell-lines could be generated to achieve reproducibility in conducting high-throughput drug screening experiments. For example, Caley et al. [92] successfully established immortalized fibroblasts derived from chronic venous leg ulcers.

#### The nature of phagocytic cargo used

The *S. aureus* strain Wood 46 attached to the pHRODO dye, used to mimic inflammation and reduced phagocytic activity in the in vitro DFU model, is a facultative anaerobe, Gram-positive human pathogen which produces toxins and lacks the cell wall-associated protein staphylococcal protein A (SpA) [93]. This latter binds tightly to immunoglobulins and, consequently, inhibits opsonization and phagocytosis effectively [94, 95]. Interestingly, SpA expression was reportedly predominant in strong and moderate biofilm-forming *S. aureus* isolates from individuals with a DFU [96]. More interestingly, exogenous application of SpA stimulated biofilm formation in SpA mutant strains, signifying that SpA does not need to be covalently attached to the cell wall for its activity.

The used *S. aureus* strain was killed using formaldehyde and alcohol desiccation. Hence, the reported findings of its phagocytic activity in this study are predominantly based on PAMP recognition and binding by THP-1-derived Mɸ, excluding the deteriorating effect of virulence factors released by viable pathogens [97]. Hence, the influence of other detrimental bacteria-induced effects on phagocytosis could not be considered. For example, urease activity, which catalyzes urea to ammonia via hydrolysis, is present in *S. aureus* and results in the pH neutralization of the acidic phagosomal microenvironment, which in turn inhibits bacterial break-down [98].

A more recent study reinforced the need to expose cells to pHRODO bioparticles for less than 6 h ideally. Decline in pHRODO fluorescence for incubation periods longer than 6 h was observed and could be a result of either complete cargo digestion or defects in acidification, rather than reduced phagocytosis. Hence, similar systems such as the more sensitive ratiometric pH-sensitive probe epHero [99], which tracks real-time changes in phagosome maturation and acidification distinctly and concurrently, could be adapted for the development of the direct co-culture-based DFU model for long-term studies.

Previous studies reported the intracellular co-localization of pHRODO *S. aureus* bioparticles within the acidic lysosomes of murine and human Mɸ [36, 100]. Hence, we recommend using the LysoTracker stain to further confirm the successful uptake of pHRODO *S. aureus* bioparticles in the in vitro wound models. Although flow cytometry is likewise relevant for verifying the successful uptake of pHRODO bioparticles intracellularly, it results tedious to conduct kinetic studies as each timepoint and condition is considered a distinct sample. Hence, the use of a high content imaging strategy results both time- and cost-effective for monitoring phagocytosis kinetics in real-time in the in vitro wound models.

#### Signalling pathway characterization

The presented work provides the foundation for better characterization of the pHRODO-based in vitro DFU model. Hence, the use of appropriate molecular biology techniques such as single-cell RNA sequencing, Western blotting and real-time-polymerase chain reaction (RT-PCR) will be helpful in identifying/studying potential pathways involved in phagocytosis.

#### Physiological microenvironment

The in vitro DFU model reported in this study was developed within a well of a 96-well plate. Cells interact on a microscale and, therefore, the in vitro DFU model can be integrated within microfluidic systems [101]. This approach will provide cost-effective and ample opportunities to control and study the effect of physiologically relevant biophysical and biochemical parameters (e.g., pressure, flowrate, shear forces, oxygen level, etc.) on phagocytosis in DFU on a physiological scale. Examples of potential studies include the chemoattraction profile of Mɸ in the presence of single/combined pHRODO-labelled viable bacteria/fungi isolated from DFU in the in vitro DFU model (e.g., *S. aureus*, *P. aeruginosa* and *C. albicans*) and real-time observation of phagocytosis kinetics.

## Conclusions

The use of pHRODO bioparticles for inducing inflammation in in vitro diseased models is believed to be the first-of-its-kind in the literature to the best of our knowledge. In this work, a preliminary and effective approach for modelling concurrently inflammation and reduced phagocytic ability in DFU in vitro was achieved using pHRODO green *S. aureus* bioparticles at reduced FBS concentration under hypoxic conditions.

For improved physiological relevance of proinflammatory stimulation and phagocytic cargo uptake modelling of DFU in vitro, given the bacterial diversity in diabetic foot infections (ranging from “mild” to “severe”) and the associated effect on wound healing outcome, present bacteria or fungi can be isolated from exudates obtained from individuals with a DFU, cultivated and, finally, conjugated with the commercially available pHRODO dye.

Phagocytosis has not been actively considered as a wound healing biomarker in DFU. However, stimulating phagocytosis has been a major drug target in treating some neurodegenerative diseases. Therefore, repurposing identified phagocytosis-stimulating drugs for neurodegenerative diseases can be explored for DFU treatment.

Hence, the resulting pHRODO labelled DFU-derived bacteria/fungi can be used for developing more physiologically relevant in vitro DFU models that can be used for high-throughput drug screening (to identify drugs that can stimulate phagocytosis and potentially contribute to DFU healing).

For inflammation resolution and to initiate neo-tissue formation, ingestion of apoptotic neutrophils by Mɸ (i.e., efferocytosis) is necessary. However, efferocytosis is significantly impaired in DFU. Hence, neutrophils from individuals with a DFU rendered apoptotic can be labelled with the commercially available pHRODO dye. The resulting bioconjugates could be used for setting up an in vitro DFU efferocytosis model for screening potential compounds to identify pro-efferocytotic hits for DFU treatment.

Finally, the high content imaging approach used in the present work has major advantages: it provides numerical values to the resulting fluorescence intensity derived from the ingested pHRODO bioparticles and the corresponding images; and it allows for testing multiple drugs at the same time.

## Supplementary Information

Below is the link to the electronic supplementary material.

Supplementary file1 (PDF 2518 KB)

## Data Availability

The data that support the findings of this study are available from the corresponding author upon request.

## Footnotes

1 ns, not significant.

## References

[1] Saeedi P et al. Global and regional diabetes prevalence estimates for 2019 and projections for 2030 and 2045: results from the International Diabetes Federation Diabetes Atlas, 9th edition. Diabetes Res Clin Pract. 2019;157. 10.1016/j.diabres.2019.107843. 10.1016/j.diabres.2019.107843 31518657

[2] Boulton AJ, Vileikyte L, Ragnarson-Tennvall G, Apelqvist J. The global burden of diabetic foot disease. Lancet. 2005;366(9498):1719–24. 10.1016/S0140-6736(05)67698-2. 16291066 10.1016/S0140-6736(05)67698-2

[3] Armstrong DG, Boulton AJM, Bus SA. Diabetic foot ulcers and their recurrence. N Engl J Med. 2017;376(24):2367–75. 10.1056/NEJMRA1615439/SUPPL_FILE/NEJMRA1615439_DISCLOSURES.PDF. 28614678 10.1056/NEJMra1615439

[4] American Diabetes Association. Diagnosis and classification of diabetes mellitus. Diabetes Care. 2014;37(Suppl 1). 10.2337/DC14-S081. 10.2337/dc14-S081 24357215

[5] American Diabetes Association. Introduction: standards of medical care in diabetes-2018. Diabetes Care. 2018;41(Suppl 1):S1–2. 10.2337/DC18-SINT01. 29222369 10.2337/dc18-Sint01

[6] Afsaneh Alavi et al. Diabetic foot ulcers: Part I. Pathophysiology and prevention. J Am Acad Dermatol. 2014;70. 10.1016/j.jaad.2013.06.055. 10.1016/j.jaad.2013.06.055 24355275

[7] Noor S, Khan RU, Ahmad J. Understanding diabetic foot infection and its management. Diabetes Metab Syndr. 2017;11(2):149–56. 10.1016/j.dsx.2016.06.023. 27377687 10.1016/j.dsx.2016.06.023

[8] Mirza R, Koh TJ. Dysregulation of monocyte/macrophage phenotype in wounds of diabetic mice. Cytokine. 2011;56(2):256–64. 10.1016/J.CYTO.2011.06.016. 21803601 10.1016/j.cyto.2011.06.016

[9] Baltzis D, Eleftheriadou I, Veves A. Pathogenesis and treatment of impaired wound healing in diabetes mellitus: new insights. Adv Ther. 2014;31(8):817–36. 10.1007/S12325-014-0140-X. 25069580 10.1007/s12325-014-0140-x

[10] Cai F, Wang P, Chen W, Zhao R, Liu Y. The physiological phenomenon and regulation of macrophage polarization in diabetic wound. Mol Biol Rep. 2023;50(11):9469–77. 10.1007/S11033-023-08782-X/FIGURES/4. 37688679 10.1007/s11033-023-08782-x

[11] Uribe-Querol E, Rosales C. Phagocytosis: our current understanding of a universal biological process. Front Immunol. 2020;11: 531655. 10.3389/FIMMU.2020.01066/BIBTEX. 10.3389/fimmu.2020.01066 PMC7280488 32582172

[12] Levin R, Grinstein S, Canton J. The life cycle of phagosomes: formation, maturation, and resolution. Immunol Rev. 2016;273(1):156–79. 10.1111/IMR.12439. 27558334 10.1111/imr.12439

[13] Aitcheson SM, Frentiu FD, Hurn SE, Edwards K, Murray RZ. Skin wound healing: normal macrophage function and macrophage dysfunction in diabetic wounds. Molecules. 2021;26(16). 10.3390/MOLECULES26164917. 10.3390/molecules26164917 PMC8398285 34443506

[14] Khanna S et al. Macrophage dysfunction impairs resolution of inflammation in the wounds of diabetic mice. PLoS One. 2010;5(3). 10.1371/JOURNAL.PONE.0009539. 10.1371/journal.pone.0009539 PMC2832020 20209061

[15] Gurjala AN, et al. Development of a novel, highly quantitative in in vivo model for the study of biofilm-impaired cutaneous wound healing. Wound Repair Regen. 2011;19(3):400–10. 10.1111/J.1524-475X.2011.00690.X. 21518094 10.1111/j.1524-475X.2011.00690.x

[16] Brandenburg KS, Calderon DF, Kierski PR, Czuprynski CJ, McAnulty JF. Novel murine model for delayed wound healing using a biological wound dressing with Pseudomonas aeruginosa biofilms. Microb Pathog. 2018;122:30–8. 10.1016/J.MICPATH.2018.05.043. 29842898 10.1016/j.micpath.2018.05.043

[17] Kasiewicz LN, Whitehead KA. Silencing TNFα with lipidoid nanoparticles downregulates both TNFα and MCP-1 in an in vitro co-culture model of diabetic foot ulcers. Acta Biomater. 2016;32:120–8. 10.1016/J.ACTBIO.2015.12.023. 26689461 10.1016/j.actbio.2015.12.023

[18] Kasiewicz LN, Whitehead KA, Kathryn Whitehead CA. Lipid nanoparticles silence tumor necrosis factor α to improve wound healing in diabetic mice. Bioeng Transl Med. 2019;4(1):75–82. 10.1002/BTM2.10123. 30680320 10.1002/btm2.10123 PMC6336737

[19] Bandyk DF. The diabetic foot: pathophysiology, evaluation, and treatment. Semin Vasc Surg. 2018;31(2–4):43–8. 10.1053/J.SEMVASCSURG.2019.02.001. 30876640 10.1053/j.semvascsurg.2019.02.001

[20] Iversen MM, et al. History of foot ulcer increases mortality among individuals with diabetes: ten-year follow-up of the Nord-Trøndelag Health Study, Norway. Diabetes Care. 2009;32(12):2193–9. 10.2337/DC09-0651. 19729524 10.2337/dc09-0651 PMC2782976

[21] Curzer HJ, Perry G, Wallace MC, Perry D. The three Rs of animal research: what they mean for the institutional animal care and use committee and why. Sci Eng Ethics. 2016;22(2):549–65. 10.1007/S11948-015-9659-8/METRICS. 26026966 10.1007/s11948-015-9659-8

[22] Maione AG, et al. Three-dimensional human tissue models that incorporate diabetic foot ulcer-derived fibroblasts mimic in in vivo features of chronic wounds. Tissue Eng Part C Methods. 2015;21(5):499. 10.1089/TEN.TEC.2014.0414. 25343343 10.1089/ten.tec.2014.0414 PMC4410281

[23] Lemarchand M, Thouin K, De Serres-Bérard T, Bellenfant S, Cadau S, Berthod F. in vitro glycation of a tissue-engineered wound healing model to mimic diabetic ulcers. Biotechnol Bioeng. 2023. 10.1002/BIT.28359. 36810698 10.1002/bit.28359

[24] Smith A, et al. A novel three-dimensional skin disease model to assess macrophage function in diabetes. Tissue Eng Part C Methods. 2021;27(2):49. 10.1089/TEN.TEC.2020.0263. 33280487 10.1089/ten.tec.2020.0263 PMC8349718

[25] Serres-Bérard D. Développement d’un modèle de peau reconstruite par génie tissulaire à partir de cellules diabétiques pour l’étude des plaies chroniques cutanées. 2019.

[26] Mashkova M, Goranov V, Mokhort T, Shyshko A, Mantachik M. Application of 3D skin culture model as a tool to study the role of immune mechanisms in chronic diabetic foot ulcers pathogenesis. Endocrine Abstracts. 2019;63. 10.1530/ENDOABS.63.GP33.

[27] Joffe AM, Bakalar MH, Fletcher DA. Macrophage phagocytosis assay with reconstituted target particles. Nature Protocols. 2020;15(7):2230–46. 10.1038/s41596-020-0330-8. 32561889 10.1038/s41596-020-0330-8

[28] Tzach-Nahman R, et al. Oral fibroblasts modulate the macrophage response to bacterial challenge. Scientific Reports. 2017;7(1):1–11. 10.1038/s41598-017-11771-3. 28912533 10.1038/s41598-017-11771-3 PMC5599598

[29] Maisonneuve L, Manoury B. in vitro and in in vivo assays to evaluate dendritic cell phagocytic capacity, in Dendritic Cells: Methods and Protocols, V. Sisirak, Ed., New York, NY: Springer US, 2023;279–288. 10.1007/978-1-0716-2938-3_20. 10.1007/978-1-0716-2938-3_20 36905524

[30] Parv K, Westerlund N, Merchant K, Komijani M, Lindsay RS, Christoffersson G. Phagocytosis and efferocytosis by resident macrophages in the mouse pancreas. Front Endocrinol (Lausanne). 2021;12:606175. 10.3389/FENDO.2021.606175/BIBTEX. 10.3389/fendo.2021.606175 PMC8185276 34113315

[31] Maguire E, Connor-Robson N, Shaw B, O’Donoghue R, Stöberl N, Hall-Roberts H. Assaying microglia functions in vitro. Cells. 2022;11(21):3414. 10.3390/CELLS11213414. 36359810 10.3390/cells11213414 PMC9654693

[32] Stöhr R, Deckers N, Schurgers L, Marx N, Reutelingsperger CP. AnnexinA5-pHrodo: a new molecular probe for measuring efferocytosis. Scientific Reports. 2018;8(1):1–9. 10.1038/s41598-018-35995-z. 30532026 10.1038/s41598-018-35995-z PMC6286334

[33] Lenzo JC, O’Brien-Simpson NM, Cecil J, Holden JA, Reynolds EC. Determination of active phagocytosis of unopsonized Porphyromonas gingivalis by macrophages and neutrophils using the pH-sensitive fluorescent dye pHrodo. Infect Immun. 2016;84(6):1753–60. 10.1128/IAI.01482-15/ASSET/2E9462A8-D5A7-42AF-8F61-D29871760369/ASSETS/GRAPHIC/ZII9990917200004.JPEG. 27021243 10.1128/IAI.01482-15 PMC4907136

[34] Mason ER, Soni DM, Chu S. Microglial phagocytosis/cell health high-content assay. Curr Protoc. 2023;3(3):e724. 10.1002/cpz1.724. 36971657 10.1002/cpz1.724 PMC10433541

[35] Nam GH, et al. An optimized protocol to determine the engulfment of cancer cells by phagocytes using flow cytometry and fluorescence microscopy. J Immunol Methods. 2019;470:27–32. 10.1016/J.JIM.2019.04.007. 31034881 10.1016/j.jim.2019.04.007

[36] Lindner B, Burkard T, Schuler M. Phagocytosis assays with different pH-sensitive fluorescent particles and various readouts. Biotechniques. 2020;68(5):245–50. 10.2144/BTN-2020-0003. 32079414 10.2144/btn-2020-0003

[37] Gradišnik L, Milojević M, Velnar T, Maver U. Isolation, characterisation and phagocytic function of human macrophages from human peripheral blood. Mol Biol Rep. 2020;47(9):6929–40. 10.1007/S11033-020-05751-6/FIGURES/4. 32876844 10.1007/s11033-020-05751-6

[38] Romero-Ramírez L, García-Rama C, Mey J. Janus kinase inhibitor brepocitinib rescues myelin phagocytosis under inflammatory conditions: in vitro evidence from microglia and macrophage cell lines. Mol Neurobiol. 2024;1–12. 10.1007/S12035-024-03963-6. 10.1007/s12035-024-03963-6 38308667

[39] Kapellos TS, et al. A novel real time imaging platform to quantify macrophage phagocytosis. Biochem Pharmacol. 2016;116:107–19. 10.1016/j.bcp.2016.07.011. 27475716 10.1016/j.bcp.2016.07.011 PMC5012892

[40] Hendrickx DAE, Schuurman KG, van Draanen M, Hamann J, Huitinga I. Enhanced uptake of multiple sclerosis-derived myelin by THP-1 macrophages and primary human microglia. J Neuroinflammation. 2014;11(1):1–11. 10.1186/1742-2094-11-64/TABLES/3. 24684721 10.1186/1742-2094-11-64 PMC4108133

[41] Zhu F, Yue W, Wang Y. The nuclear factor kappa B (NF-κB) activation is required for phagocytosis of staphylococcus aureus by RAW 264.7 cells. Exp Cell Res. 2014;327(2):256–63. 10.1016/J.YEXCR.2014.04.018. 24798100 10.1016/j.yexcr.2014.04.018

[42] Chaiwut R, Kasinrerk W. Very low concentration of lipopolysaccharide can induce the production of various cytokines and chemokines in human primary monocytes. BMC Res Notes. 2022;15(1):42. 10.1186/s13104-022-05941-4. 35144659 10.1186/s13104-022-05941-4 PMC8832778

[43] El-Zayat SR, Sibaii H, Mannaa FA. Toll-like receptors activation, signaling, and targeting: an overview. Bull Natl Res Cent. 2019;43(1):1–12.

[44] Connor SM et al. GW5074 increases microglial phagocytic activities: potential therapeutic direction for Alzheimer’s disease. Front Cell Neurosci. 2022;16. [Online]. Available: https://www.frontiersin.org/articles/10.3389/fncel.2022.894601 10.3389/fncel.2022.894601 PMC9169965 35677758

[45] Nizami S, Hall-Roberts H, Warrier S, Cowley SA, Di Daniel E. Microglial inflammation and phagocytosis in Alzheimer’s disease: potential therapeutic targets. Br J Pharmacol. 2019;176(18):3515–32. 10.1111/bph.14618. 30740661 10.1111/bph.14618 PMC6715590

[46] Salter MW, Stevens B. Microglia emerge as central players in brain disease. Nat Med. 2017;23(9):1018–27. 10.1038/nm.4397. 28886007 10.1038/nm.4397

[47] Mescher AL. Junqueira's Basic Histology. 17th ed. 2023.

[48] Gordon S. Phagocytosis: an immunobiologic process. Immunity. 2016;44(3):463–75. 10.1016/J.IMMUNI.2016.02.026. 26982354 10.1016/j.immuni.2016.02.026

[49] Ozleyen A, Yilmaz YB, Tumer TB. Dataset on the differentiation of THP-1 monocytes to LPS inducible adherent macrophages and their capacity for NO/iNOS signaling. Data Brief. 2021;35:106786. 10.1016/j.dib.2021.106786. 33553532 10.1016/j.dib.2021.106786 PMC7851796

[50] Yang J, et al. Role of MCP-1 in tumor necrosis factor-α-induced endothelial dysfunction in type 2 diabetic mice. Am J Physiol Heart Circ Physiol. 2009;297(4):H1208. 10.1152/AJPHEART.00396.2009. 19666844 10.1152/ajpheart.00396.2009 PMC2770760

[51] Kanda H, et al. MCP-1 contributes to macrophage infiltration into adipose tissue, insulin resistance, and hepatic steatosis in obesity. J Clin Invest. 2006;116(6):1494–505. 10.1172/JCI26498. 16691291 10.1172/JCI26498 PMC1459069

[52] Álvaro-Afonso FJ, García-Álvarez Y, Tardáguila-García A, García-Madrid M, López-Moral M, Lázaro-Martínez JL. Bacterial diversity and antibiotic resistance in patients with diabetic foot osteomyelitis. Antibiotics. 2023;12(2):212. 10.3390/ANTIBIOTICS12020212. 36830123 10.3390/antibiotics12020212 PMC9951858

[53] Franklin RA. Fibroblasts and macrophages: collaborators in tissue homeostasis. Immunol Rev. 2021;302(1):86–103. 10.1111/IMR.12989. 34101202 10.1111/imr.12989

[54] Zhou X, et al. Microenvironmental sensing by fibroblasts controls macrophage population size. Proc Natl Acad Sci U S A. 2022;119(32):e2205360119. 10.1073/PNAS.2205360119/SUPPL_FILE/PNAS.2205360119.SAPP.PDF. 35930670 10.1073/pnas.2205360119 PMC9371703

[55] Liu J, et al. NF-κB activation is critical for bacterial lipoprotein tolerance-enhanced bactericidal activity in macrophages during microbial infection. Scientific Reports. 2017;7(1):1–15. 10.1038/srep40418. 28079153 10.1038/srep40418 PMC5227741

[56] Essen BioScience, “Kinetic analysis of microglial function and morphology.” Accessed: May 11, 2023. [Online]. Available: https://www.news-medical.net/whitepaper/20200714/Kinetic-Analysis-of-Microglial-Function-and-Morphology.aspx

[57] Ramirez-Acuña JM, et al. Diabetic foot ulcers: current advances in antimicrobial therapies and emerging treatments. Antibiotics. 2019;8(4):193. 10.3390/ANTIBIOTICS8040193. 31652990 10.3390/antibiotics8040193 PMC6963879

[58] Dasari N, et al. Healing, inflammation, and fibrosis: updates in diabetic wound healing, inflammation, and scarring. Semin Plast Surg. 2021;35(3):153. 10.1055/S-0041-1731460. 34526862 10.1055/s-0041-1731460 PMC8432997

[59] Voza FA, et al. Fibroblasts in diabetic foot ulcers. Int J Molecul Sci. 2024;25(4):2172. 10.3390/IJMS25042172. 10.3390/ijms25042172 PMC10889208 38396848

[60] Lee DY, et al. Analysis of commercial fetal bovine serum (FBS) and its substitutes in the development of cultured meat. Food Res Int. 2023;174:113617. 10.1016/j.foodres.2023.113617. 37986472 10.1016/j.foodres.2023.113617

[61] Sierra A, Abiega O, Shahraz A, Neumann H. Janus-faced microglia: beneficial and detrimental consequences of microglial phagocytosis. Front Cell Neurosci. 2013;7, [Online]. Available: https://www.frontiersin.org/journals/cellular-neuroscience/articles/10.3389/fncel.2013.00006 10.3389/fncel.2013.00006 PMC3558702 23386811

[62] Wu S, Romero-Ramírez L, Mey J. Taurolithocholic acid but not tauroursodeoxycholic acid rescues phagocytosis activity of bone marrow-derived macrophages under inflammatory stress. J Cell Physiol. 2022;237(2):1455–70. 10.1002/JCP.30619. 34705285 10.1002/jcp.30619 PMC9297999

[63] Sivagnanam V, Zhu X, Schlichter LC. Dominance of E. coli phagocytosis over LPS in the inflammatory response of microglia. J Neuroimmunol. 2010;227(1–2):111–9. 10.1016/J.JNEUROIM.2010.06.021. 20655116 10.1016/j.jneuroim.2010.06.021

[64] Radzieta M, et al. A multiomics approach to identify host-microbe alterations associated with infection severity in diabetic foot infections: a pilot study. NPJ Biofilms Microbiomes. 2021;7(1):29. 33753735 10.1038/s41522-021-00202-x PMC7985513

[65] Pettersson US, et al. Increased recruitment but impaired function of leukocytes during inflammation in mouse models of type 1 and type 2 diabetes. PLoS ONE. 2011;6(7):e22480. 21799868 10.1371/journal.pone.0022480 PMC3143146

[66] Rich J, Lee JC. The pathogenesis of Staphylococcus aureus infection in the diabetic NOD mouse. Diabetes. 2005;54(10):2904–10. 16186391 10.2337/diabetes.54.10.2904

[67] Wang X, Yuan C-X, Xu B, Yu Z. Diabetic foot ulcers: classification, risk factors and management. World J Diabetes. 2022;13(12):1049. 10.4239/WJD.V13.I12.1049. 36578871 10.4239/wjd.v13.i12.1049 PMC9791567

[68] Yotsu RR, et al. Comparison of characteristics and healing course of diabetic foot ulcers by etiological classification: neuropathic, ischemic, and neuro-ischemic type. J Diabetes Complications. 2014;28(4):528–35. 10.1016/J.JDIACOMP.2014.03.013. 24846054 10.1016/j.jdiacomp.2014.03.013

[69] Schumacker PT. Hypoxia-inducible factor-1 (HIF-1). Crit Care Med. 2005;33(12):S423–5. 16340411 10.1097/01.ccm.0000191716.38566.e0

[70] Nizet V, Johnson RS. Interdependence of hypoxic and innate immune responses. Nat Rev Immunol. 2009;9(9):609–17. 19704417 10.1038/nri2607 PMC4343208

[71] Rodrigues M, Kosaric N, Bonham CA, Gurtner GC. Wound healing: a cellular perspective. Physiol Rev. 2019;99(1):665–706. 30475656 10.1152/physrev.00067.2017 PMC6442927

[72] Bento CF, et al. The chaperone-dependent ubiquitin ligase CHIP targets HIF-1α for degradation in the presence of methylglyoxal. PLoS ONE. 2010;5(11):e15062. 21124777 10.1371/journal.pone.0015062 PMC2993942

[73] Anand RJ, et al. Hypoxia causes an increase in phagocytosis by macrophages in a HIF-1α-dependent manner. J Leukoc Biol. 2007;82(5):1257–65. 10.1189/JLB.0307195. 17675562 10.1189/jlb.0307195

[74] Zhang Y, et al. Hypoxia-inducible factor-1α promotes macrophage functional activities in protecting hypoxia-tolerant large yellow croaker (Larimichthys crocea) against Aeromonas hydrophila infection. Front Immunol. 2024;15:1410082. 39156889 10.3389/fimmu.2024.1410082 PMC11327042

[75] Chen YC, Chou WY, Fu TC, Wang JS. Effects of normoxic and hypoxic exercise training on the bactericidal capacity and subsequent apoptosis of neutrophils in sedentary men. Eur J Appl Physiol. 2018;118(9):1985–95. 10.1007/s00421-018-3935-7. 29987365 10.1007/s00421-018-3935-7

[76] Vivekanand Datta C, et al. HIF-1α expression regulates the bactericidal capacity of phagocytes. J Clin Invest. 2005;115(7):1806–15. 16007254 10.1172/JCI23865 PMC1159132

[77] Fazli M, et al. Nonrandom distribution of Pseudomonas aeruginosa and Staphylococcus aureus in chronic wounds. J Clin Microbiol. 2009;47(12):4084. 10.1128/JCM.01395-09. 19812273 10.1128/JCM.01395-09 PMC2786634

[78] Dhekane R, Mhade S, Kaushik KS. Adding a new dimension: Multi-level structure and organization of mixed-species Pseudomonas aeruginosa and Staphylococcus aureus biofilms in a 4-D wound microenvironment. Biofilm. 2022;4:100087. 10.1016/J.BIOFLM.2022.100087. 36324526 10.1016/j.bioflm.2022.100087 PMC9618786

[79] Redel H, et al. Quantitation and composition of cutaneous microbiota in diabetic and nondiabetic men. J Infect Dis. 2013;207:1105–14. 10.1093/infdis/jit005. 23300163 10.1093/infdis/jit005 PMC3583274

[80] Jneid J, Lavigne JP, La Scola B, Cassir N. The diabetic foot microbiota: a review. Hum Microb J. 2017;5–6:1–6. 10.1016/j.humic.2017.09.002.

[81] Zhang S-S, Tang Z-Y, Fang P, Qian H-J, Xu L, Ning G. Nutritional status deteriorates as the severity of diabetic foot ulcers increases and independently associates with prognosis. Exp Ther Med. 2013;5(1):215–22. 23251271 10.3892/etm.2012.780 PMC3524099

[82] Raja JM, Maturana MA, Kayali S, Khouzam A, Efeovbokhan N. Diabetic foot ulcer: a comprehensive review of pathophysiology and management modalities. World J Clin Cases. 2023;11(8):1684. 10.12998/WJCC.V11.I8.1684. 36970004 10.12998/wjcc.v11.i8.1684 PMC10037283

[83] Lecube A, Pachón G, Petriz J, Hernández C, Simó R. Phagocytic activity is impaired in type 2 diabetes mellitus and increases after metabolic improvement. PLoS ONE. 2011;6(8):e23366. 21876749 10.1371/journal.pone.0023366 PMC3158070

[84] Xu Y, et al. The impact of inflammatory biomarkers on amputation rates in patients with diabetic foot ulcers. Int Wound J. 2024;21(4):e14827. 38522433 10.1111/iwj.14827 PMC10961172

[85] Escobar IE, Possamai Rossatto FC, Kim SM, Kang MH, Kim W, Mylonakis E. Repurposing kinase inhibitor Bay 11–7085 to combat Staphylococcus aureus and Candida albicans biofilms. Front Pharmacol. 2021;12. 10.3389/FPHAR.2021.675300/FULL. 10.3389/fphar.2021.675300 PMC8133364 34025434

[86] Eming SA, Krieg T, Davidson JM. Inflammation in wound repair: molecular and cellular mechanisms. J Invest Dermatol. 2007;127(3):514–25. 10.1038/SJ.JID.5700701. 17299434 10.1038/sj.jid.5700701

[87] Forrester MA, et al. Similarities and differences in surface receptor expression by THP-1 monocytes and differentiated macrophages polarized using seven different conditioning regimens. Cell Immunol. 2018;332:58–76. 30077333 10.1016/j.cellimm.2018.07.008 PMC7611637

[88] Liu B-F, et al. Low phagocytic activity of resident peritoneal macrophages in diabetic mice: relevance to the formation of advanced glycation end products. Diabetes. 1999;48(10):2074–82. 10512376 10.2337/diabetes.48.10.2074

[89] Rungratanawanich W, Qu Y, Wang X, Essa MM, Song B-J. Advanced glycation end products (AGEs) and other adducts in aging-related diseases and alcohol-mediated tissue injury. Exp Mol Med. 2021;53(2):168–88. 33568752 10.1038/s12276-021-00561-7 PMC8080618

[90] Maione AG, et al. Altered ECM deposition by diabetic foot ulcer-derived fibroblasts implicates fibronectin in chronic wound repair. Wound Repair Regen. 2016;24(4):630. 10.1111/WRR.12437. 27102877 10.1111/wrr.12437 PMC5500637

[91] Liang L, et al. Integrative analysis of miRNA and mRNA paired expression profiling of primary fibroblast derived from diabetic foot ulcers reveals multiple impaired cellular functions. Wound Repair and Regeneration. 2016;24(6):943–53. 10.1111/WRR.12470. 27607190 10.1111/wrr.12470 PMC5470742

[92] Caley M, Wall IB, Peake M, Kipling D, Giles P, Thomas DW, Stephens P. Development and characterisation of a human chronic skin wound cell line-towards an alternative for animal experimentation. Int J Mol Sci. 2018;19(4):1001. Accessed: May 26, 2024. [Online]. 10.3390/ijms19041001 10.3390/ijms19041001 PMC5979489 29584680

[93] BacDive. Staphylococcus aureus Wood 46, Wood | DSM 20491, ATCC 10832, CCM 2351, NCTC 7121, CIP 52.1 | BacDiveID:14490. Accessed: May 31, 2024. [Online]. Available: https://bacdive.dsmz.de/strain/14490

[94] Sulica A, Medesan C, Laky M, Onica D, Sjoquist J, Ghetie V. Effect of protein A of Staphylococcus aureus on the binding of monomeric and polymeric IgG to Fc receptor-bearing cells. Immunology. 1979;38(1):173, Accessed: Sep. 22, 2024. [Online]. Available: /pmc/articles/PMC1457909/?report=abstract PMC1457909 511216

[95] Uribe-Quero E, Rosales C. Control of phagocytosis by microbial pathogens. Front Immunol. 2017;8:302803. 10.3389/FIMMU.2017.01368/BIBTEX. 10.3389/fimmu.2017.01368 PMC5660709 29114249

[96] Mamdoh H, et al. Clinical and bacteriological analyses of biofilm-forming Staphylococci isolated from diabetic foot ulcers. Infect Drug Resist. 2023;16:1737–50. 10.2147/IDR.S393724. 36999125 10.2147/IDR.S393724 PMC10046123

[97] Herzberg C, van Meegen EN, van Hasselt JGC. Interplay of virulence factors shapes ecology and treatment outcomes in polymicrobial infections. Math Biosci. 2024;377:109293. 39245301 10.1016/j.mbs.2024.109293

[98] Bore E, Langsrud S, Langsrud Ø, Rode TM, Holck A. Acid-shock responses in Staphylococcus aureus investigated by global gene expression analysis. Microbiology (N Y). 2007;153(7):2289–303. 10.1099/MIC.0.2007/005942-0. 10.1099/mic.0.2007/005942-0 17600073

[99] Singh S, Bensalem J, Hein LK, Casey A, Mäkinen VP, Sargeant TJ. epHero – a tandem-fluorescent probe to track the fate of apoptotic cells during efferocytosis. Cell Death Discovery. 2024;10(1):1–11. 10.1038/s41420-024-01952-1. 38632247 10.1038/s41420-024-01952-1 PMC11024195

[100] Chen M-S, Lin W-C, Yeh H-T, Hu C-L, Sheu S-M. Propofol specifically suppresses IL-1β secretion but increases bacterial survival in Staphylococcus aureus-infected RAW264.7 cells. Mol Cell Biochem. 2018;449(1–2):117–25. 10.1007/s11010-018-3348-2. 29667111 10.1007/s11010-018-3348-2 PMC6223810

[101] Ejiugwo M, Rochev Y, Gethin G, O’Connor G. toward developing immunocompetent diabetic foot ulcer-on-a-chip models for drug testing. Tissue Eng Part C Methods. 2021;27(2):77–88. 10.1089/TEN.TEC.2020.0331. 33406980 10.1089/ten.TEC.2020.0331

[102] Sinha A, Jebrail MJ, Kim H, Patel KD, Branda SS. A versatile automated platform for micro-scale cell stimulation experiments. J Vis Exp. 2013;(78):50597. 10.3791/50597 10.3791/50597 PMC3854557 23962881

