# Supplementary material for: The development of a direct co-culture-based model for diabetic foot ulcer mimicking inflammation and impaired phagocytosis
Source: In Vitro Model. 2025 Apr 14;4(2):111–29. doi: 10.1007/s44164-024-00080-5 (PMC12283541; doi:10.1007/s44164-024-00080-5)
Supplement: Supplementary file 1 — Supplementary file1 (PDF 2518 KB) [file 44164_2024_80_MOESM1_ESM.pdf]

Supporting Information

The Development of a Direct Co-culture Based Model for Diabetic Foot Ulcer Mimicking Inflammation and Impaired Phagocytosis

Mirella Ejiugwo[1], Yury Rochev[1], Georgina Gethin[2], Gerard O'Connor[1]

[1] *School of Natural Sciences, University of Galway, Galway, Ireland*

[2] *School of Nursing and Midwifery, University of Galway, Galway, Ireland*

pHRODO^{TM} bioparticles - mechanism of action

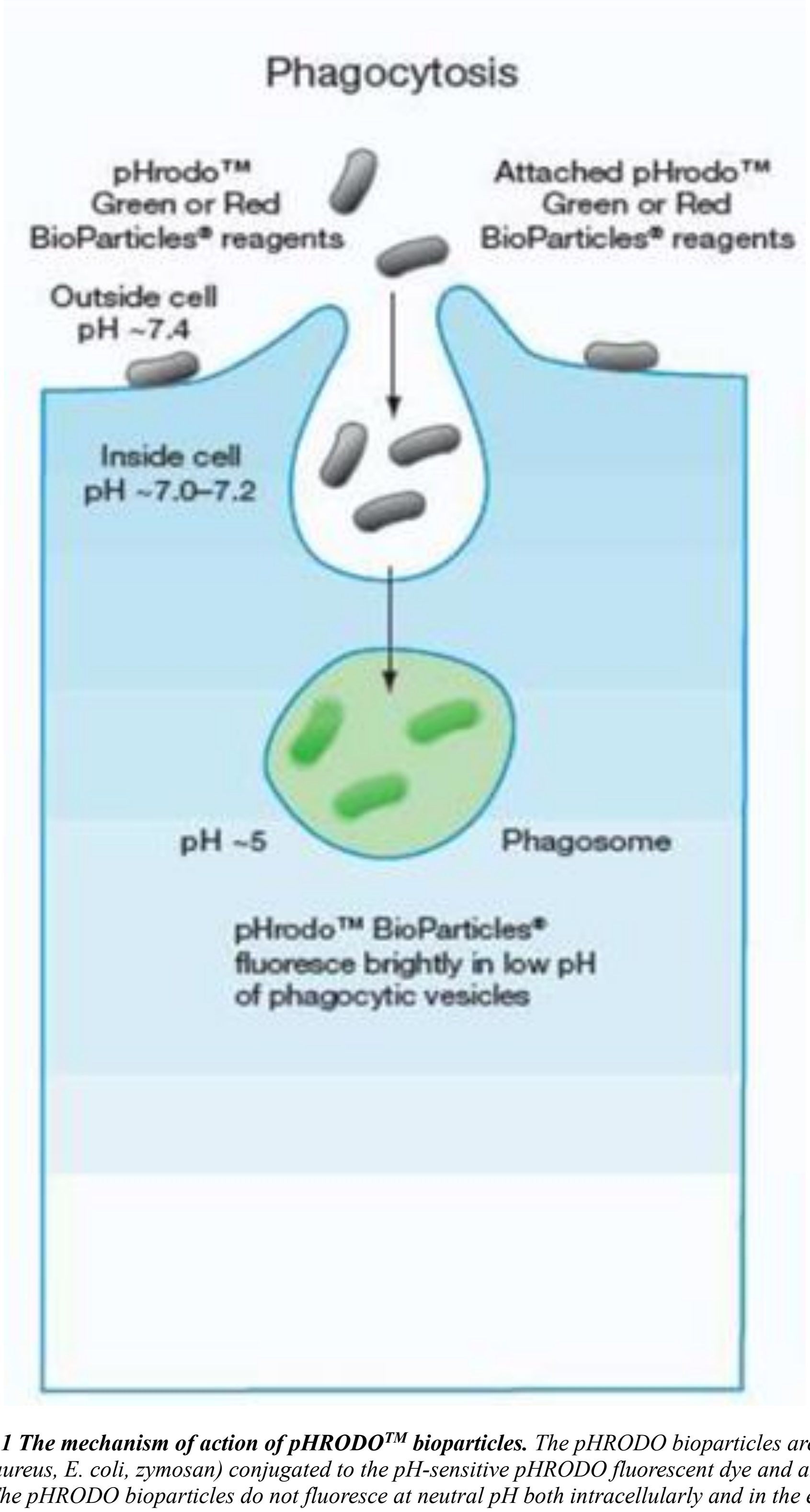

Supplementary Fig. 1 The mechanism of action of pHRODO^{TM} bioparticles. *The pHRODO bioparticles are chemically-killed microorganisms (S. aureus, E. coli, zymosan) conjugated to the pH-sensitive pHRODO fluorescent dye and are used as phagocytic cargos. The pHRODO bioparticles do not fluoresce at neutral pH both intracellularly and in the extracellular space. The pHRODO bioparticles fluoresce only when they are located within the acidic phagocytic vesicles in the cytosol such as phagosomes and phagolysosomes. Source: Thermo Fisher Scientific*

Timepoint assay to identify optimum period of proinflammatory mediator stimulation for concurrent release of TNF-α and MCP-1

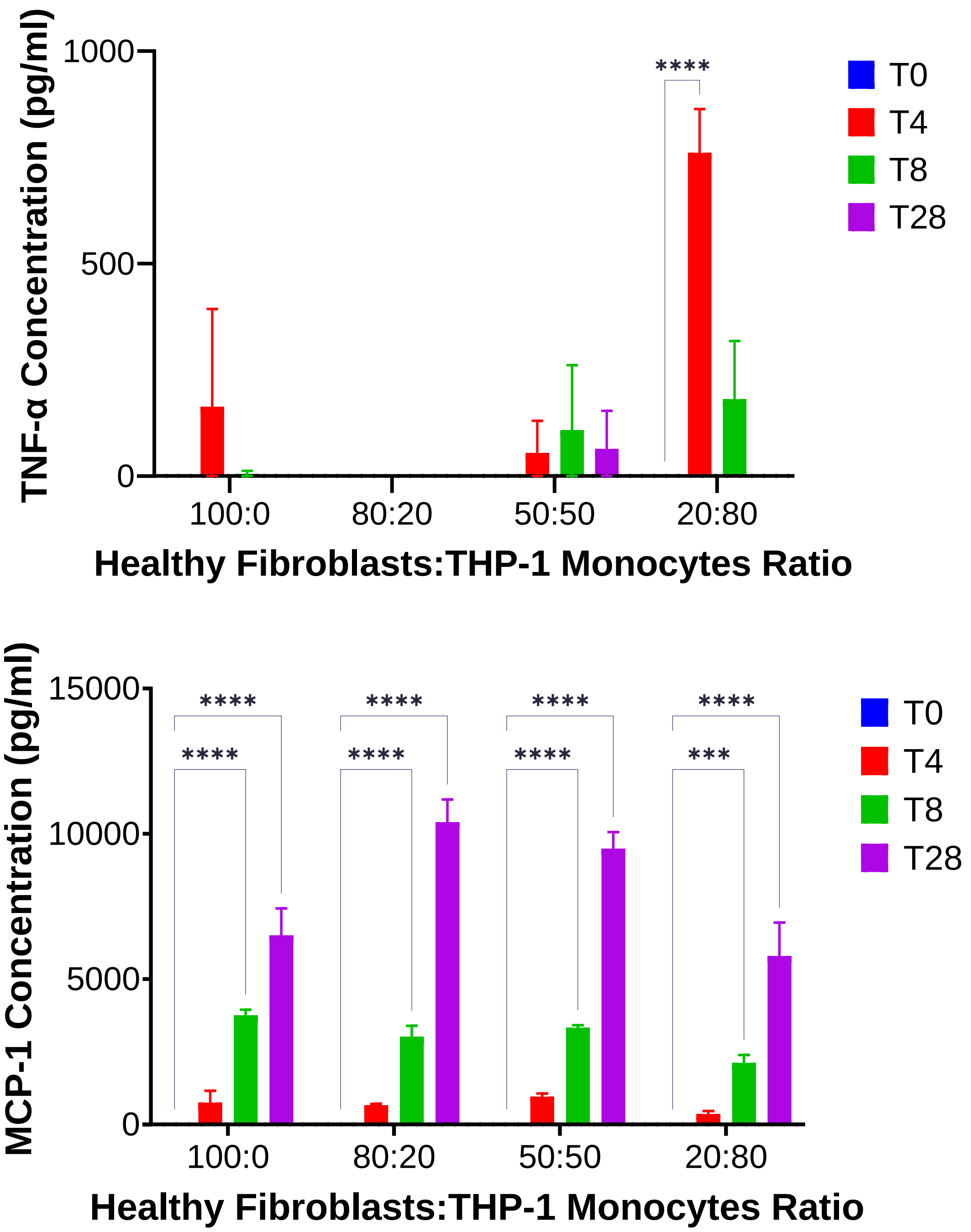

Supplementary Fig. 2 Proinflammatory mediator release following LPS exposure. *Graphs showing (a) TNF-α and (b) MCP-1 release during the 28-hour timepoint assay by different ratios of healthy dermal fibroblasts:THP-1 monocytes direct co-cultures under normoxia. Cytokine release by direct co-cultures of diabetic fibroblasts: THP-1 monocytes are expected to be relatively more pro-inflammatory than the healthy direct co-culture. T0 = before LPS stimulation; T4 = 4 hours post-LPS stimulation; T8 = 8 hours post-LPS stimulation; T28 = 28 hours post-LPS stimulation. Data points are represented as mean values ± SD. Statistical significance (p value) = 0.1234(ns), 0.0332 (*), 0.0021(**), 0.0002(***) and 0.0001(****)*

The effect of different concentrations of pHRODO bioparticles on the metabolic activity of direct co-cultures of healthy/diabetic fibroblasts and THP-1 monocytes

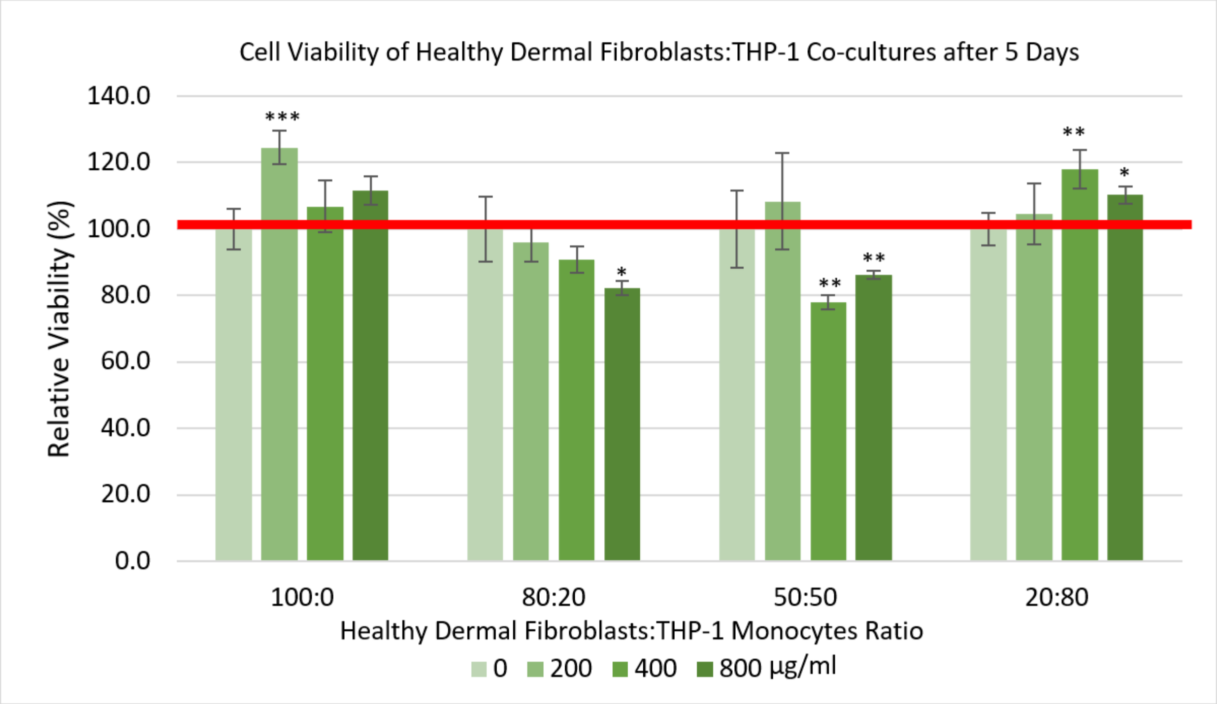

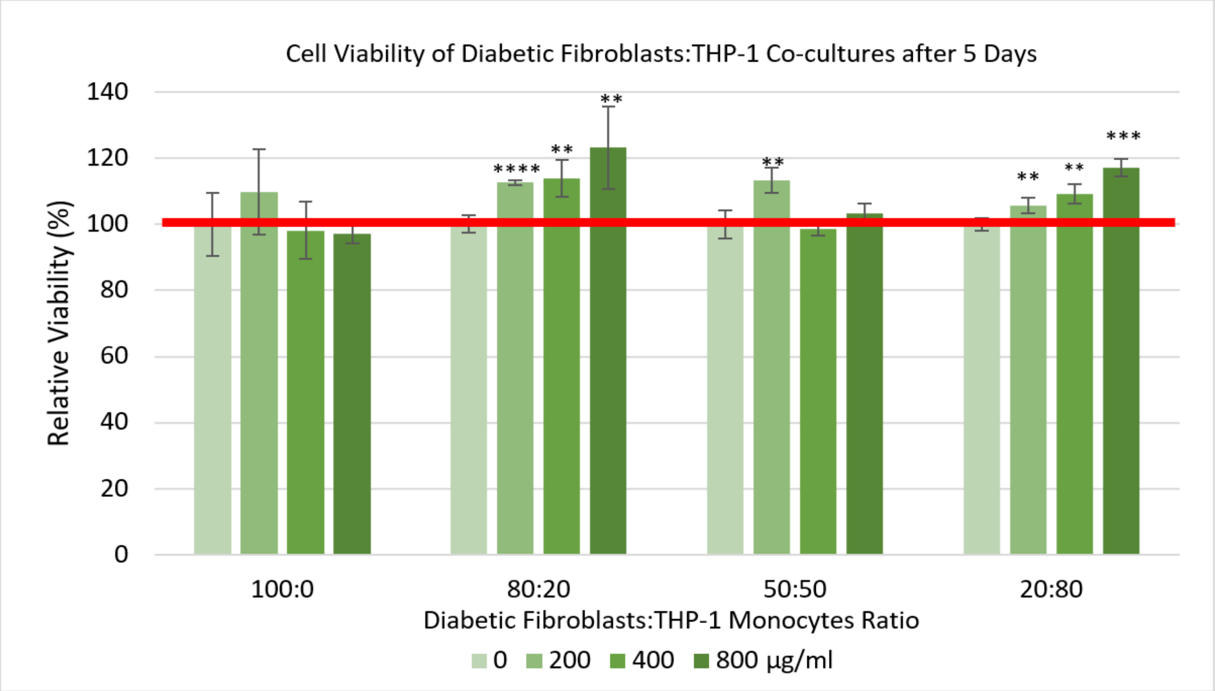

***Supplementary Fig. 3 The effect of pHRODO bioparticle concentration on metabolic activity**. Cell viability upon four-hour exposure to different pHRODO green E. coli bioparticle concentrations of (a) healthy dermal fibroblasts:THP-1 monocytes and (b) diabetic dermal fibroblast:THP-1 monocytes after five days of direct co-culture are shown. The red line intersects the measurements derived from the untreated groups. Data points are represented as mean values ± SD. Statistical significance (p value) = 0.1234(ns), 0.0332 (*), 0.0021(**), 0.0002(***) and 0.0001(****)*

TNF-α and MCP-1 release by *in vitro* DFU and healthy wound models following exposure to different pHRODO bioparticles

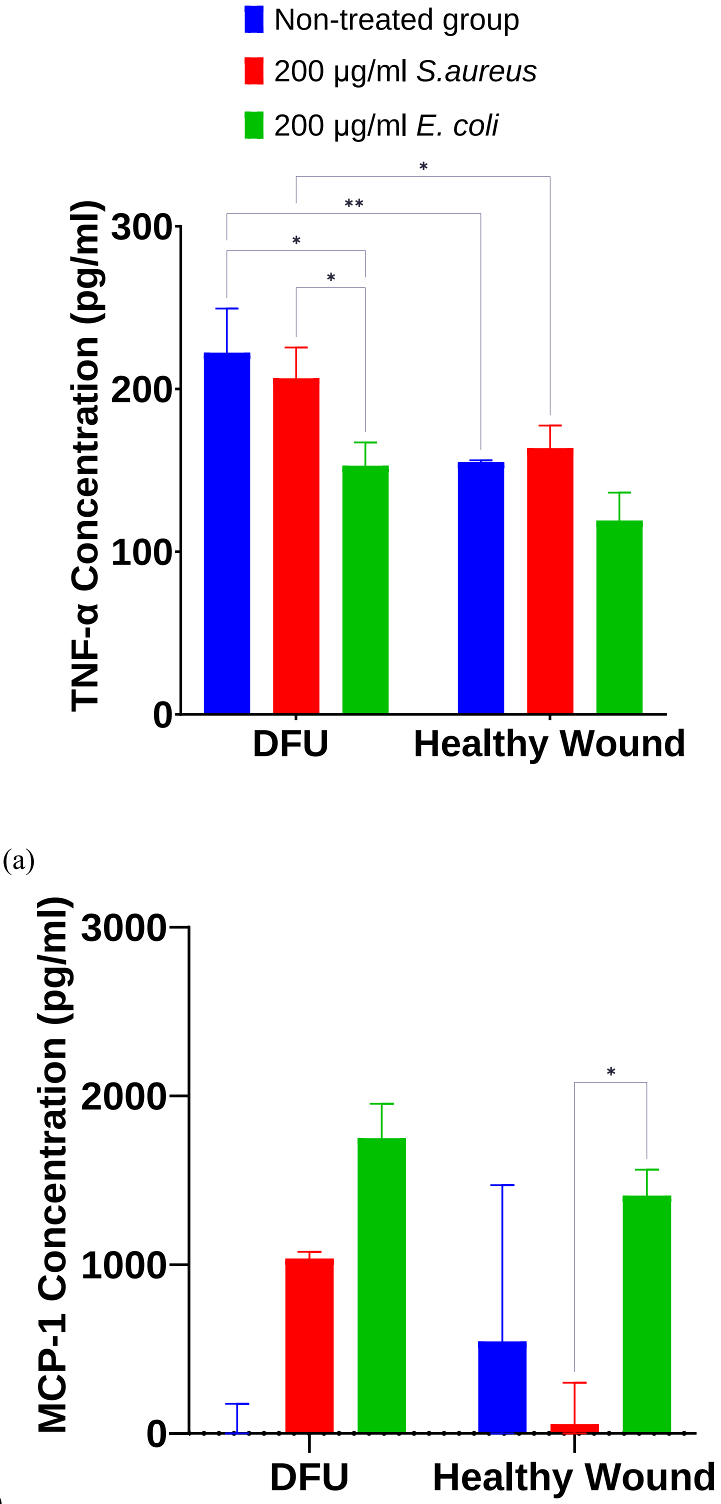

(a)

(b)

Supplementary Fig. 4 Proinflammatory mediator release following pHRODO bioparticle exposure. *TNF-α and (b) MCP-1 release after 4 hours of pHRODO bioparticle exposure under normoxia using CCM containing 2% FBS*

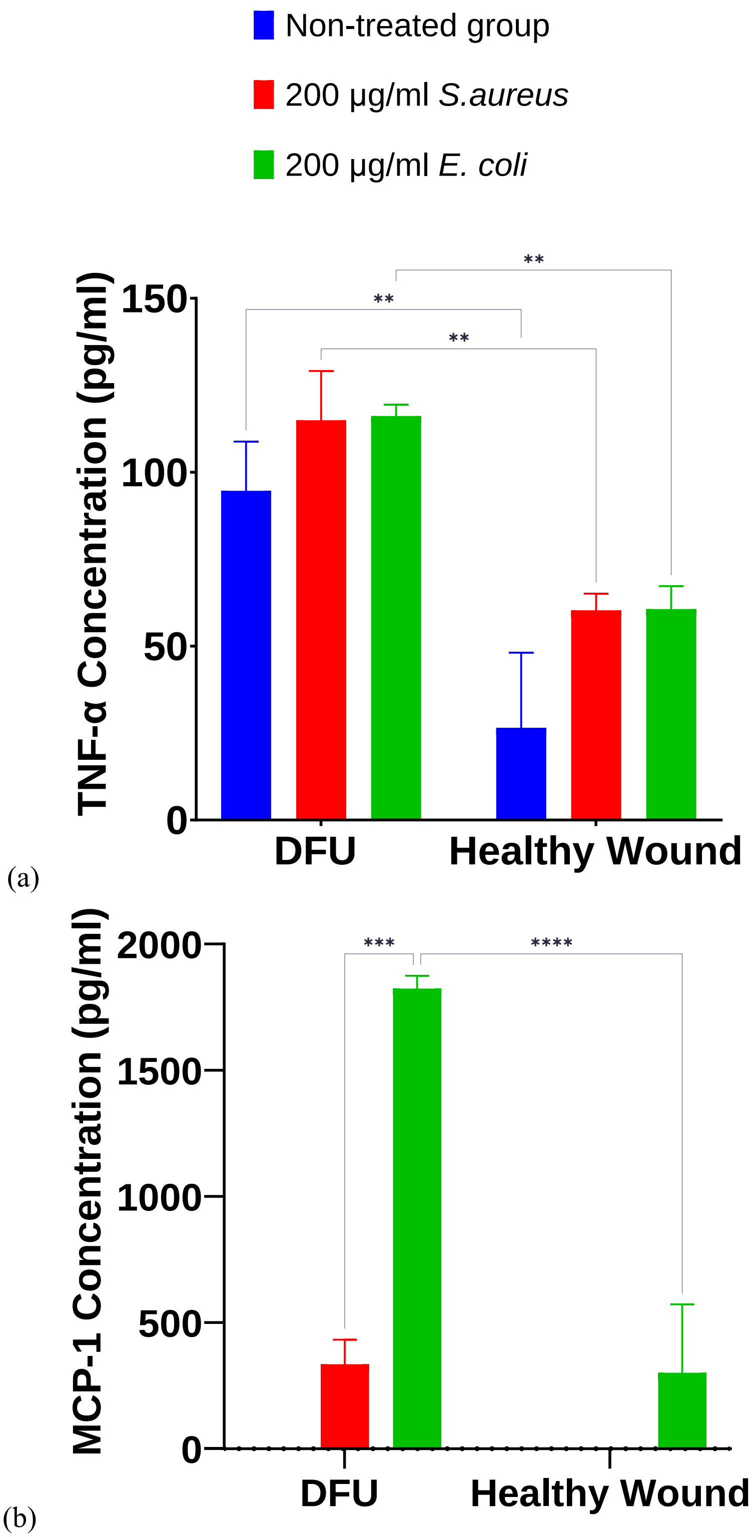

Supplementary Fig. 5 Proinflammatory mediator release following pHRODO bioparticle exposure. *(a) TNF- α and (b) MCP-1 release after 4 hours of pHRODO bioparticle exposure under hypoxia using CCM containing 2% FBS*

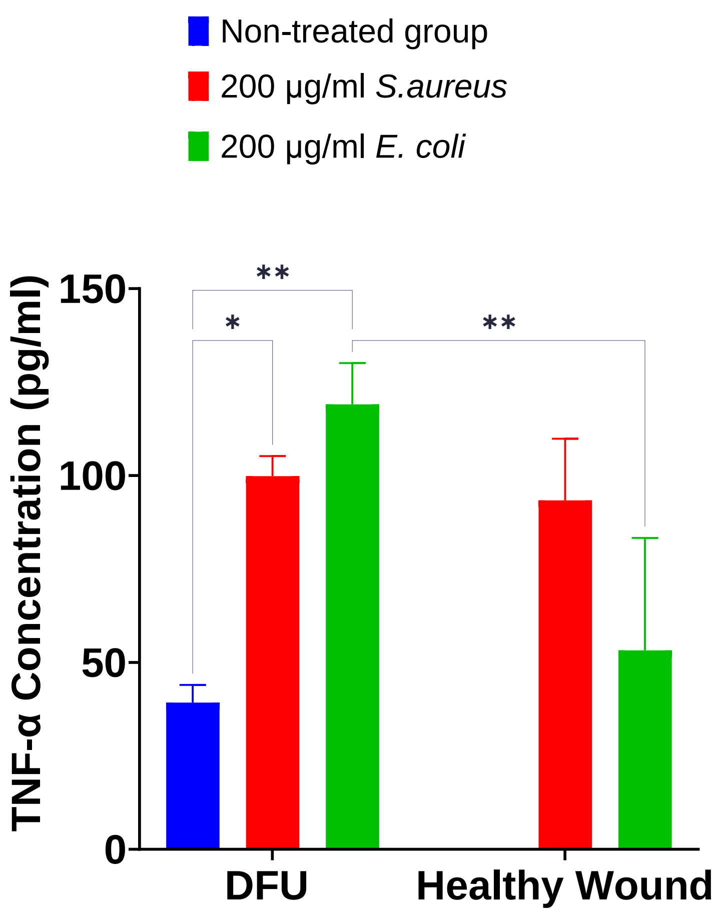

(a)

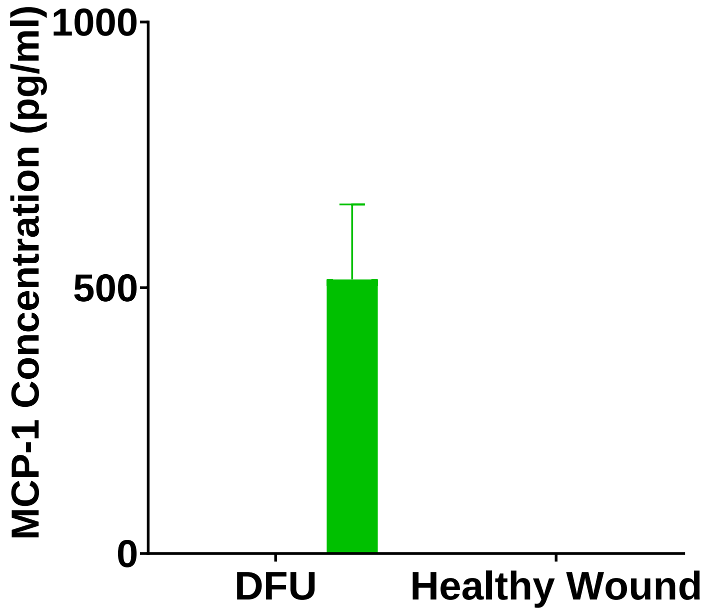

(b)

Supplementary Fig. 6 Proinflammatory mediator release following pHRODO bioparticle exposure. *(a) TNF- α and (b) MCP-1 release after 4 hours of pHRODO bioparticle exposure under hypoxia using CCM containing 10% FBS*

The uptake of pHRODO red *E. coli* bioparticles under normoxia

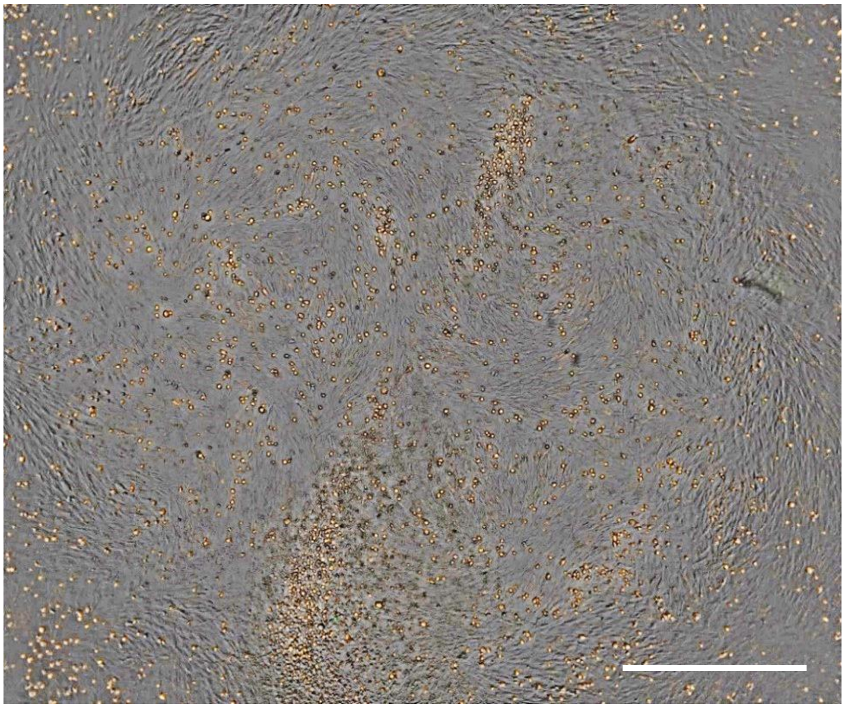

(a)

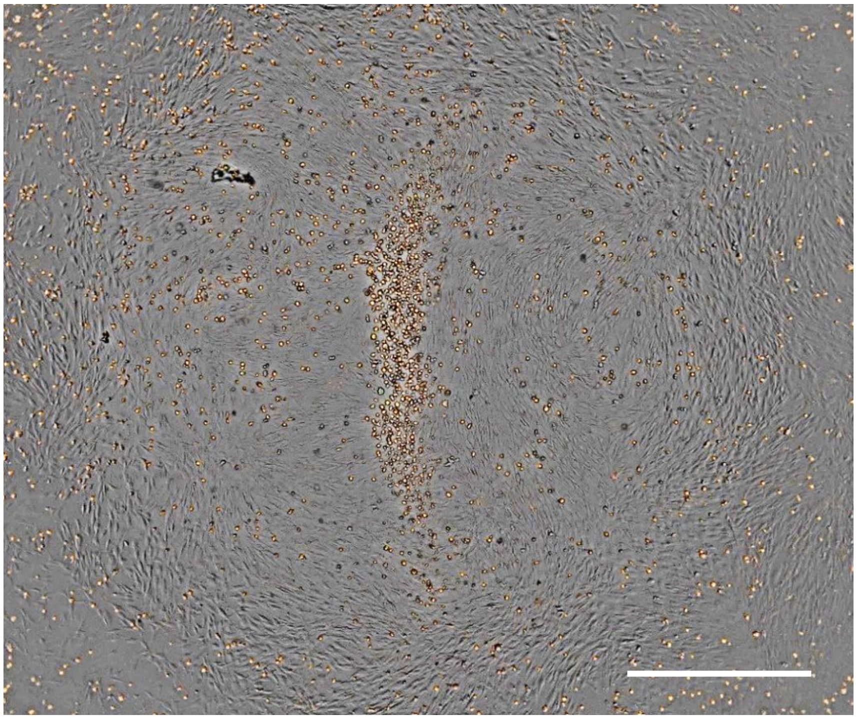

(b)

Supplementary Fig. 7 Uptake of pHRODO red E. coli bioparticles under normoxia. *Representative images of the uptake of pHRODO red E. coli bioparticles after four hours under normoxia using 10% FBS in (a) the DFU co-culture model and (b) the healthy wound co-culture model(4x magnification). Scalebar = 100 µm*

The uptake of pHRODO red *E. coli* bioparticles under hypoxia

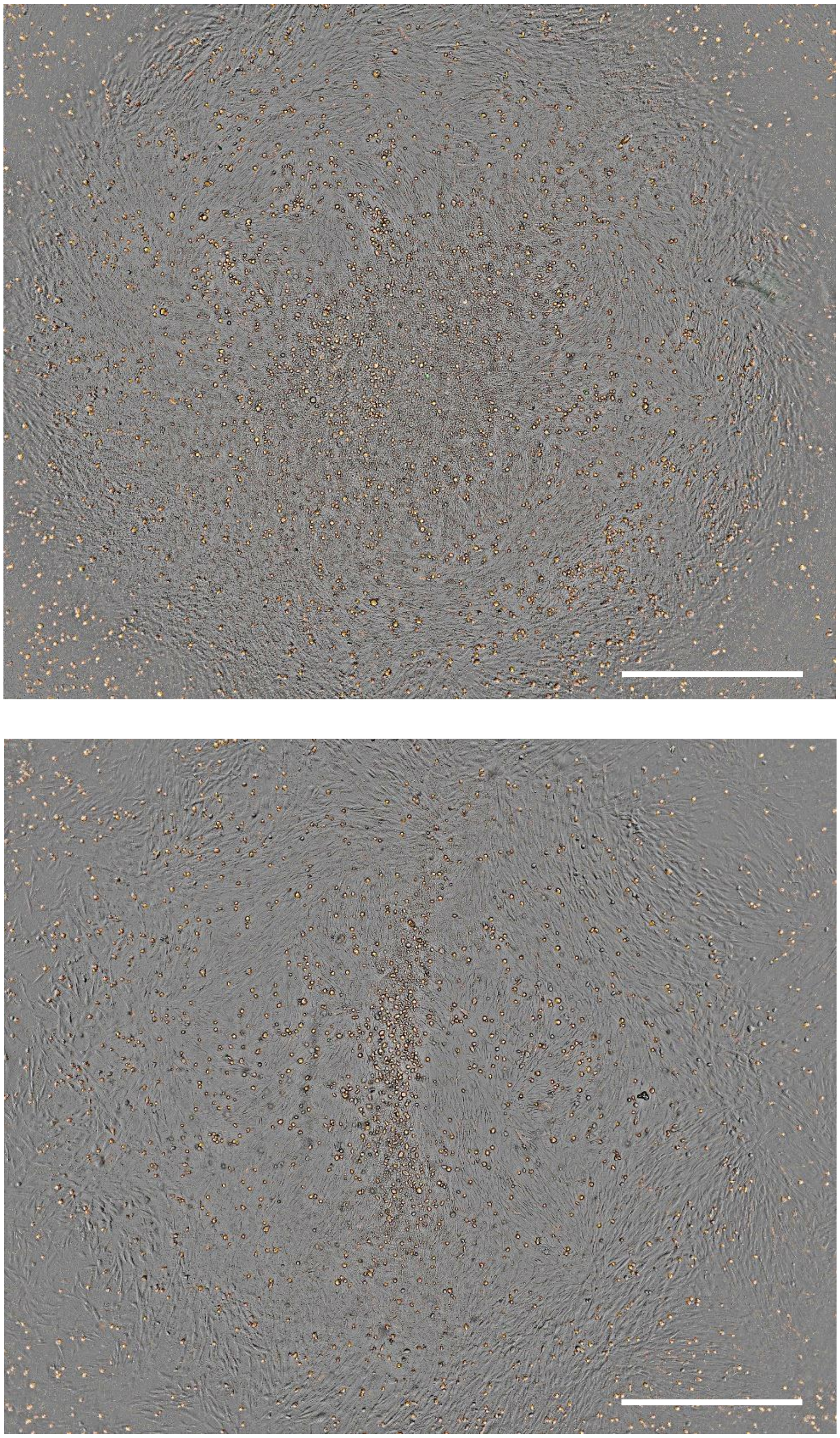

(a)

(b)

Supplementary Fig. 8 Uptake of pHRODO red E. coli bioparticles under hypoxia. *Representative images of the uptake of pHRODO red E. coli bioparticles after four hours under hypoxia using 10% FBS in (a) the DFU co-culture model and (b) the healthy wound co-culture model are shown (4x magnification). Scalebar = 100 µm*

The uptake of pHRODO green *S. aureus* bioparticles under normoxia

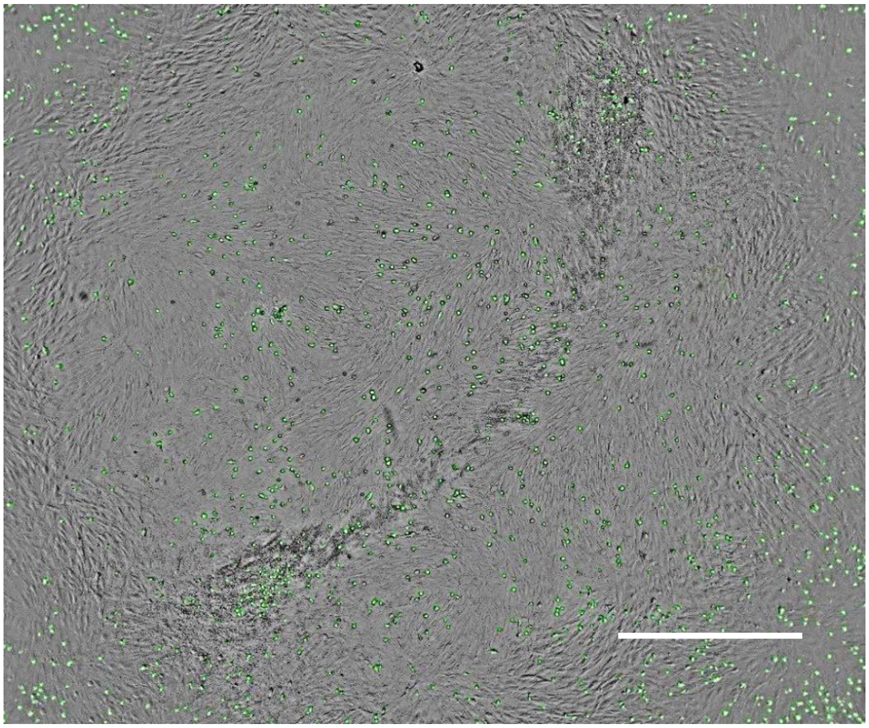

(a)

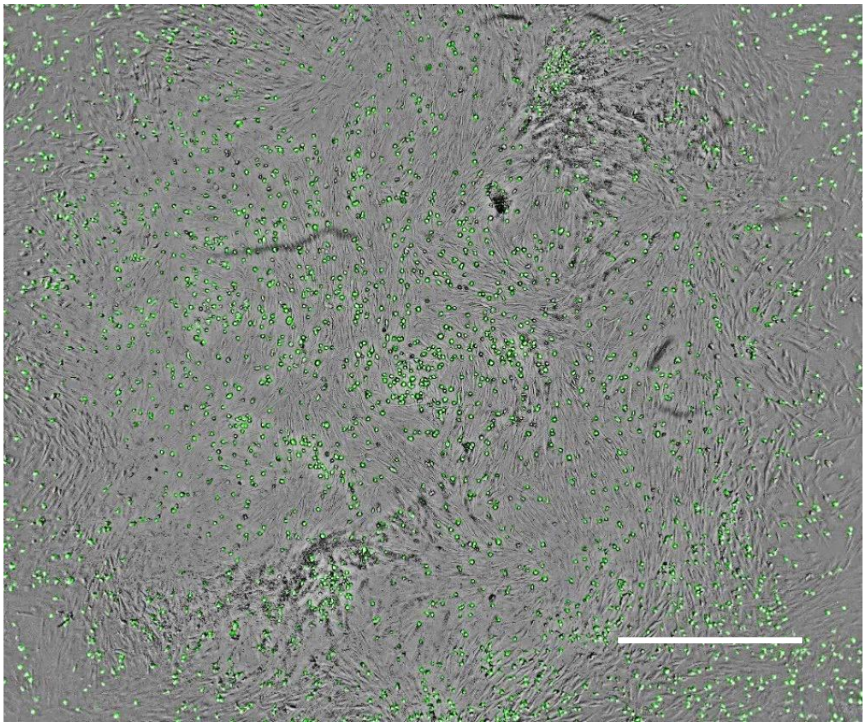

(b)

Supplementary Fig. 9 Uptake of pHRODO green S. aureus bioparticles under normoxia. *Representative images of the uptake of pHRODO green S. aureus bioparticles after four hours under normoxia using 10% FBS in (a) the DFU co-culture model and(b) the healthy wound co-culture model (4x magnification). Scalebar =100 µm*

The uptake of pHRODO green *S. aureus* bioparticles under hypoxia

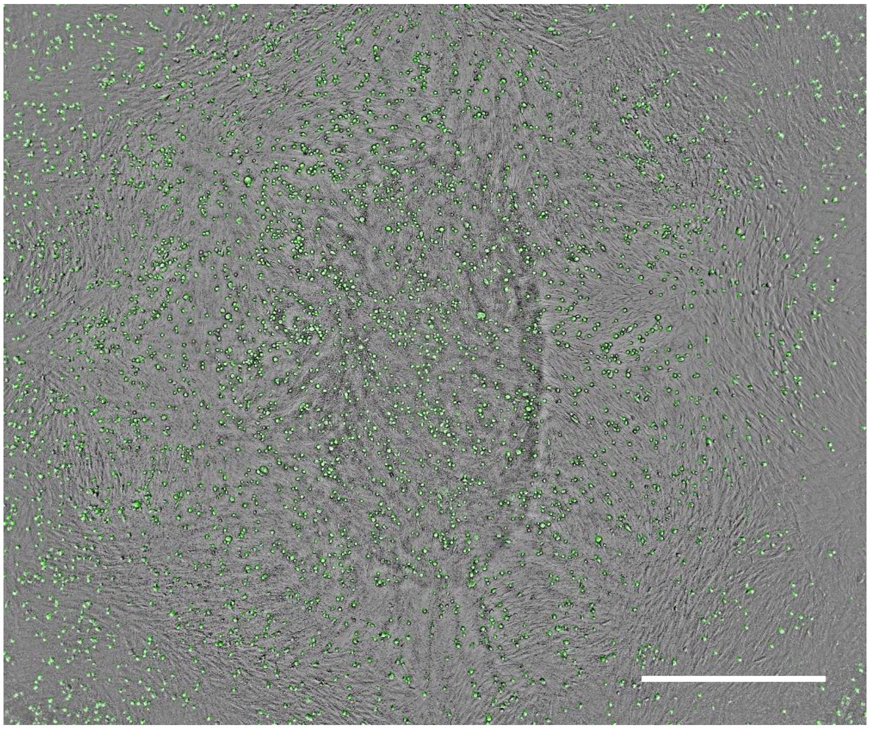

(a)

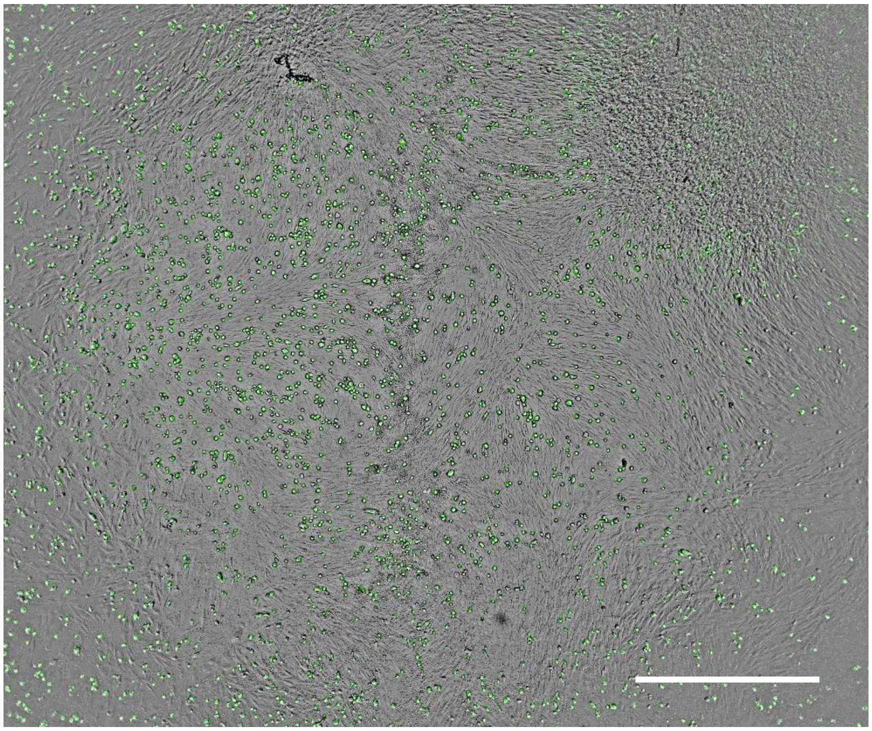

(b)

Supplementary Fig. 10 Uptake of pHRODO green S. aureus bioparticles under hypoxia. *Representative images of the uptake of pHRODO green S. aureus bioparticles after four hours under hypoxia using 10% FBS in (a) the DFU co-culture model and(b) the healthy wound co-culture model (4x magnification). Scalebar =100 µm*
